# Targeting mitochondrial and cytosolic substrates of TRIT1 isopentenyltransferase: Specificity determinants and tRNA-i^6^A37 profiles

**DOI:** 10.1371/journal.pgen.1008330

**Published:** 2020-04-23

**Authors:** Abdul Khalique, Sandy Mattijssen, Alexander F. Haddad, Shereen Chaudhry, Richard J. Maraia

**Affiliations:** 1 Intramural Research Program of the *Eunice Kennedy Shriver* National Institute of Child Health and Human Development, of the National Institutes of Health, Bethesda, Maryland, United States of America; 2 Commissioned Corps, United States Public Health Service, Rockville, Maryland, United States of America; Ohio State University, UNITED STATES

## Abstract

The tRNA isopentenyltransferases (IPTases), which add an isopentenyl group to *N*^*6*^ of A37 (i^**6**^A37) of certain tRNAs, are among a minority of enzymes that modify cytosolic and mitochondrial tRNAs. Pathogenic mutations to the human IPTase, TRIT1, that decrease i^**6**^A37 levels, cause mitochondrial insufficiency that leads to neurodevelopmental disease. We show that TRIT1 encodes an amino-terminal mitochondrial targeting sequence (MTS) that directs mitochondrial import and modification of mitochondrial-tRNAs. Full understanding of IPTase function must consider the tRNAs selected for modification, which vary among species, and in their cytosol and mitochondria. Selection is principally via recognition of the tRNA A36-A37-A38 sequence. An exception is unmodified tRNA^**Trp**^CCA-A37-A38 in *Saccharomyces cerevisiae*, whereas tRNA^**Trp**^CCA is readily modified in *Schizosaccharomyces pombe*, indicating variable IPTase recognition systems and suggesting that additional exceptions may account for some of the tRNA-i^**6**^A37 paucity in higher eukaryotes. Yet TRIT1 had not been characterized for restrictive type substrate-specific recognition. We used i^**6**^A37-dependent tRNA-mediated suppression and i^**6**^A37-sensitive northern blotting to examine IPTase activities in *S*. *pombe* and *S*. *cerevisiae* lacking endogenous IPTases on a diversity of tRNA-A36-A37-A38 substrates. Point mutations to the TRIT1 MTS that decrease human mitochondrial import, decrease modification of mitochondrial but not cytosolic tRNAs in both yeasts. TRIT1 exhibits clear substrate-specific restriction against a cytosolic-tRNA^**Trp**^CCA-A37-A38. Additional data suggest that position 32 of tRNA^**Trp**^CCA is a conditional determinant for substrate-specific i^**6**^A37 modification by the restrictive IPTases, Mod5 and TRIT1. The cumulative biochemical and phylogenetic sequence analyses provide new insights into IPTase activities and determinants of tRNA-i^**6**^A37 profiles in cytosol and mitochondria.

## Author summary

tRNA isopentenyltransferases (IPTases) are tRNA modification enzymes that are conserved in bacteria and eukaryotes. They add an isopentenyl group to the Adenosine base at position 37, adjacent to the anticodon of specific subsets of tRNAs that decode codons that begin with Uridine. This modification stabilizes the otherwise weak adjacent codon-anticodon base pair and increases the efficiency of decoding of the corresponding codons of the genetic code. IPTases belong to a group of enzymes that modify both cytoplasmic and mitochondrial tRNAs of eukaryotic cells. Interestingly, during evolution there were changes in the way that IPTases are targeted to mitochondria as well as changes in the relative numbers and identities of IPTase tRNA substrates in the cytoplasm vs. mitochondria. The latter is consistent with phenotypic consequences of IPTase deficiencies in fission and budding yeasts, and mammals. Pathogenic mutations to human IPTase (TRIT1) cause mitochondrial insufficiency and neurodevelopmental disease, principally due to decreased modification of the mt-tRNA substrates. In this study, we identify the way human TRIT1 is targeted to mitochondria. We also show that TRIT1 exhibits a tRNA anticodon identity-specific substrate sensitivity. The work leads to new understanding of the IPTases and the variable anticodon identities of their tRNA substrates found throughout nature.

## Introduction

45 different eukaryotic cytoplasmic (cy-) and about half as many mitochondrial (mt-) tRNAs contain unique sets of modifications that are considered in two groups [1–3]; those in the body or core, which contribute to folding and/or stability, and those in the anticodon loop (ACL) which contribute to mRNA decoding by cy- and mt- ribosomes. Modifications in the anticodon stem loop (ASL) are more concentrated and diverse than those in the tRNA body. One of these is isopentenylation of *N*^***6***^ of adenosine, found only on tRNAs that decode UNN codons, at position 37 (i^**6**^A37) directly 3' to the anticodon. Bacterial i^**6**^A37 is hypermodified to 2-methylthio-*N*^**6**^-isopentenyl-A37 (ms^**2**^i^**6**^A37), whereas eukaryotic cy-tRNA-i^**6**^A37 is not. Also, while the mt-tRNAs of *S*. *cerevisiae* and *S*. *pombe* are not hypermodified, mammalian mt-tRNAs i^**6**^A37 are [4–6] (Table 1).

**Table 1:** tRNA-i6A37 identity variation among species, cytoplasm and mitochondria.

tRNA anticodon (as set by DNA)	*E*. *coli* *MiaA*	*S*. *pombe* *tit1*^*+*^	*S*. *cerevisiae* *MOD5*	*human or bovine TRIT1*
cy-Ser GGA	A_37_			
cy-Ser AGA		**i**^**6**^**A**_**37**_	**i**^**6**^**A**_**37**_	**i**^**6**^**A**_**37**_
cy-Ser CGA	**ms**^**2**^**i**^**6**^**A**_**37**_	**i**^**6**^**A**_**37**_	**i**^**6**^**A**_**37**_	**i**^**6**^**A**_**37**_
cy-Ser UGA	**ms**^**2**^**i**^**6**^**A**_**37**_	**i**^**6**^**A**_**37**_	**i**^**6**^**A**_**37**_	**i**^**6**^**A**_**37**_
cy-Ser^SeC^ UCA	**i**^**6**^**A**_**37**_			**i**^**6**^**A**_**37**_
cy-Tyr GUA	**ms**^**2**^**i**^**6**^**A**_**37**_	**i**^**6**^**A**_**37**_	**i**^**6**^**A**_**37**_	m^**1**^G_37_
cy-Cys GCA	**ms**^**2**^**i**^**6**^**A**_**37**_	(G)	**i**^**6**^**A**_**37**_	m^**1**^G_37_
cy-Trp CCA	**ms**^**2**^**i**^**6**^**A**_**37**_	**i**^**6**^**A**_**37**_	**A**_**37**_	m^**1**^G_37_
cy-Phe GAA	**ms**^**2**^**i**^**6**^**A**_**37**_	yW_37_	yW_37_	yW_37_
cy-Leu CAA	**ms**^**2**^**i**^**6**^**A**_**37**_	(G)	m^**1**^G_37_	m^**1**^G_37_
cy-Leu UAA	**ms**^**2**^**i**^**6**^**A**_**37**_	(G)	m^**1**^G_37_	m^**1**^G_37_
No. isoacceptors^**@**^/ cy-tRNAs-i^**6**^A37	6/9	3/5	3/5	1/4
^#^mt-Trp CCA		**i**^**6**^**A**_**37**_		
^#^mt-Trp UCA			**i**^**6**^**A**_**37**_	**ms**^**2**^**i**^**6**^**A**_**37**_
mt-Tyr GUA		(G)	**i**^**6**^**A**_**37**_	**ms**^**2**^**i**^**6**^**A**_**37**_
mt-Ser UGA		(G)	m^**1**^G_37_	**ms**^**2**^**i**^**6**^**A**_**37**_
mt-Phe GAA		(G)	m^**1**^G_37_	**ms**^**2**^**i**^**6**^**A**_**37**_
mt-Cys GCA		**i**^**6**^**A**_**37**_	**i**^**6**^**A**_**37**_	**i**^**6**^**A**_**37**_
mt-Leu UAA		(A)*	m^**1**^G_37_	A*
No. isoacceptors/ mt-tRNAs-i^6^A37		2/2	3/3	5/5
**Total number of tRNAs-i**^**6**^**A37**	**9**	**7**	**8**	**9**

^@^Isoacceptors are tRNAs with different anticodons that are acylated with the same amino acid.

The blank white rectangle spaces denote that the tRNA is not expressed in this species [71].

^**#**^Position 34 of *S*. *pombe* mt-tRNA^**Trp**^ is encoded as C [84] whereas in *S*. *cerevisiae* [50, 104], bovine and human [105], it is encoded to be U, and was determined to be 5-taurinomethyluridine (tm^**5**^U) in bovine [4], and cmnm^**5**^U in *S*. *cerevisiae* [92] (see tRNAmodViz server [83]).

^*^A designates an A37 that is not in an A36-A37-A38 context, containing C38 [4] instead of A as is found in its *E*. *coli* counterpart.

The pattern listed in the human or bovine column is also the same for *C*. *elegans*, *Drosophila melanogaster*, *Xenopus tropicalis*, *Gallus gallus*, *and Mus musculus* [71].

yW = ybutosine.

All entries with subscript 37 have been verified as modified or unmodified; the A_37_ in the *S. cerevisiae MOD5* column indicates an exceptional unmodified A37 in a A36-A37-A38 context (see text).

Entries in parentheses show the DNA-encoded nucleotide but have not been validated as modified or not in RNA [4, 43, 59, 60].

*S*. *cerevisiae* mt-tRNA^**Trp**^ i^**6**^A37 was reported by [50] and cited in [51] as i^**6**^A37 or ms^**2**^i^**6**^A37.

*Sup-3e* [106] (encodes cy-tRNA^**Ser**^UCA, also referred to as pSer-tRNA [33, 107]) contains mcm^**5**^U [94] as does mammalian selenocysteine-inserting cy-tRNA^**Ser[Sec]**^UCA [95]. Also see *E*. *coli* tRNA^**Ser[Sec]**^UCA [108].

The mt-tRNAs^**Trp**^ recognize 2 sense codons in budding yeast and animal mitochondria [109] [4].

We note that several yeast species encode cy-tRNAs^**Trp**^ with G37 ([71], see below), and for the two mitochondrial genomes we examined, *Kluyveromyces lactis* and *Yarrowia lipolytica*, both encode mt-tRNAs^**Trp**^UCA [110, 111].

i^**6**^A37 increases tRNA affinity for the ribosome, increases anticodon-codon pairing occupancy, can activate mRNA decoding and decrease frameshifting (reviewed in [7, see 8]). ASL modifications contribute to "circuits" [9], in which one is prerequisite for formation of others, and whose disruptions can cause disease [10, 11]. I^**6**^A37 is required for m^**3**^C32 formation on the tRNAs^**Ser**^ that decode UCN codons [12, 13].

Among the numerous enzymes that modify cy- and mt-tRNAs, there are three that recognize the ACLs of their substrates and whose mutations lead to neurodevelopmental disorders. These are TRIT1 (tRNA isopentenyl transferase-1)[14, 15], THG1L (tRNA-histidine guanylyltransferase-1) [16, 17], and TRMT5 (tRNA methyltransferase-5)[18–20]. Thg1 recognizes the anticodon identity element to distinguish its substrate from other tRNAs, even though it modifies a distant site on the tRNA [21]. The Trm5 and IPTase enzymes differentially modify position 37 of numerous tRNAs, using distinct recognition mechanisms [22–24]. As detailed below, it is unclear how IPTases recognize and distinguish among some of their substrates.

IPTases have been conserved through evolution. tRNA i^**6**^A37 modification can be functionally complemented in *S*. *cerevisiae* lacking its IPTase, Mod5, by cDNAs encoding IPTases from human (TRIT1), silkworm (GRO-1), *S*. *pombe* (Tit1), plants or even moss that produce prokaryote-type IPTase [25–29]. Complementation is often shown using tRNA-mediated suppression (TMS) which reflects decoding of an mRNA premature stop codon by a suppressor-tRNA^**Tyr**^UUA that requires i^**6**^A37 for activity. A comparable TMS system in *S*. *pombe* is mediated by suppressor-tRNA^**Ser**^UCA [30–33]. In both systems, TMS can be readily monitored by a red-white colony assay. Full length TRIT1 (1–467) cloned from human cDNA is active for TMS in *S*. *cerevisiae* [25]. Human TRIT1 also complements tRNA^**Ser**^UCA-i^**6**^A37-mediated TMS in *S*. *pombe* although the *TRIT1-R323Q* mutation associated with neurodevelopmental disease is largely inactive in the same assay [14].

The single gene-encoded Mod5 localizes to nuclei, nucleoli, cytoplasm and mitochondria [34–36]. Nuclear Mod5 is involved in tRNA gene-mediated silencing [37, 38]; its localization [36] is consistent with this and early tRNA processing [39, 40]. For some tRNAs, A36-A37-A38 is interrupted by an intron, and in yeast where tRNA splicing occurs on the surface of mitochondria, tRNA nuclear export is prerequisite to i^**6**^A37 formation [2, 41, also see 42]. A Mod5 nuclear localization signal [36] is C-terminal to the eukaryote-specific IPTase Zn-finger motif [7] that binds the tRNA backbone [24], and is required for the activity of the *S*. *Pombe* IPTase Tit1 [43].

**Fig 1:**
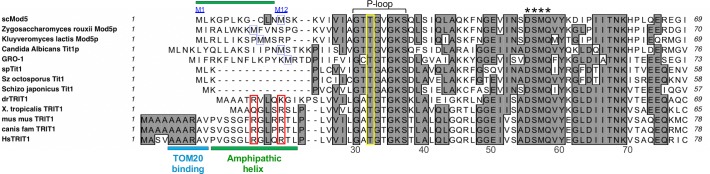
N-termini sequence alignment of representatives of phylogenetic groups of IPTases. Sequences of the N-termini of IPTases. scMod5: *Saccharomyces cerevisiae*; GRO-1: IPTase from *C*. *elegans*; spTit1: *Schizosaccharomyces (Sz) pombe*; Sz *octosporus*; Sz *japonicus*; drTRIT1: *D*. *rerio*: *Danio rerio; mus mus*: *Mus musculus; canis fam*: *Canis familiaris; X*. *tropicalis*: *Xenopus tropicalis; Hs*: *Homo sapiens*. Numerals below the alignment show human amino acid numbering. The region between M1 and M12 of Mod5 that is required for mitochondrial targeting is indicated by a green rectangle above [34, 35]. The second methionines that may be used as alternative translation start sites for cytoplasmic localization are boxed in blue. The invariant Threonine within the conserved P-loop that functions in catalysis is in the yellow rectangle. Asterisks indicate the conserved DSMQ sequence that forms a network of contacts that mediate A37 recognition; the D of which acts as a general base for catalysis [24] and the M of which is M57 of TRIT1(57–467). As predicted by MitoFates [53], the TOM20-binding site of human TRIT1 is underlined with a blue bar and the amphipathic helix of TRIT1 and Mod5 are indicated with green bars. The two key basic residues in the conserved amphipathic helix of mammalian MTS that were mutated in human TRIT1 are in red rectangles.

I^**6**^A37 modification of mt-tRNA in the mitochondrial matrix is a critical TRIT1 function [14]. Pathophysiology of *TRIT1*-*R323Q*-associated neurodevelopmental disease is attributable to deficient mt-tRNA modification and consequent impairment of mitochondrial mRNA translation, even though cy-tRNAs^**Ser**^ are also hypomodified [14]. Though *S*. *cerevisiae* and *C*. *elegans* use alternative translation initiation to target their IPTases to mitochondria [34, 35, 44], the mechanism used by TRIT1 would appear to differ (Fig 1) yet had not been subjected to experimental analysis. In the first part of this study we examine mitochondrial targeting of TRIT1 in human cells. We then extend this to TRIT1 mitochondrial function in *S*. *pombe* and *S*. *cerevisiae* model systems. Later, we examine modification of a diversity of tRNA substrates by TRIT1, Mod5 and Tit1, and the extent to which substrate-specific negative determinants may contribute to tRNAs-i^**6**^A37 profile variation (below).

A conserved positive determinant of all known IPTase substrates is the A36-A37-A38 recognition sequence required for i^**6**^A37 formation. Structures of bacterial and *S*. *cerevisiae* IPTases bound to their substrates and biochemical studies show that they directly recognize the ACLs of their target tRNAs and mediate i^**6**^A37 formation using dimethylallyl-pyrophosphate (DMAPP) as the isopentenyl donor [24, 45–48]. Intriguingly, despite these conserved features and effects of i^**6**^A37 on translation, variation exists in the tRNAs-i^**6**^A37 profiles among species (Table 1). Although i^**6**^A37 occurs widely on bacterial tRNAs for UNN codons, fewer cy-tRNAs bear i^**6**^A37 in *S*. *pombe*, *S*. *cerevisiae* and human (Table 1). tRNA-i^**6**^A37 identities also vary between mitochondria and cytosol, within and among species. Better understanding of a basis of this variation is a goal of our study.

Table 1 emphasizes that while a single gene encodes the cytoplasmic and mitochondrial IPTases of the species listed, the cy- and mt- tRNA-i^**6**^A37 profiles differ. Specifically, while TRIT1 modifies only tRNAs^**Ser**^ in cytosol, it modifies five tRNA types in mitochondria (Table 1).

Table 1 represents a phylogenetic analysis that reveals substitution of A37 with G as a major source of variability among cy-tRNAs-i^**6**^A37, especially eukaryotes. tRNA^**Ser**^GGA is only one of ten A36-A37-A38 tRNAs in *E*. *coli* that lack i^**6**^A37; weak ASL base pairs decrease its substrate activity for MiaA [49]. Lack of i^**6**^A37 on *S*. *cerevisiae* cy-tRNA^**Trp**^CCA-A37-A38 is attributable to its CCA ACL as determined *in vivo* and *in vitro*, the latter with recombinant Mod5 on cell-derived cy-tRNA^**Trp**^ and synthetic ASL substrates [43]. By contrast, *S*. *pombe* Tit1, which has an extended anticodon recognition loop relative to Mod5, readily modifies *S*. *pombe* cy-tRNA^**Trp**^CCA and the same ASL substrates [43]. Hence, Mod5 is denoted as a restrictive, and Tit1 as a nonrestrictive type eukaryotic IPTase. It is notable that while A37 of cy-tRNA^**Trp**^CCA-A37-A38 is unmodified in *S*. *cerevisiae*, the A37 of mt-tRNA^**Trp**^UCA-A37-A38 was reported as modified with i^**6**^A [50, see 51, Table 1, below]. Accordingly, the *S*. *cerevisiae* tRNA-i^**6**^A37 identity profile is defined in part by the A36-A37-A38 motif and in part by a distinct characteristic of its IPTase. Yet, the significance of the cy-tRNA^**Trp**^CCA exception in which the AC is a negative determinant in the presence of A36-A37-A38 was enigmatic and deserved further consideration.

Thus, it might appear that tRNA-i^**6**^A37 variation can be accounted for by a simple two component model, i) direct IPTase recognition of A36-A37-A38, ii) A37G substitution in tRNAs that decode UNN codons, and an exception, the unmodified A37 of cy-tRNA^**Trp**^CCA-A37-A38 in *S*. *cerevisiae*. The exception is intriguing because its proposed mechanism involves the AC recognition loop of Mod5, mutagenesis of which altered the hierarchical substrate preference for tRNA^**Ser**^, tRNA^**Tyr**^, and tRNA^**Cys**^ [43]. By this model, cy-tRNA^**Trp**^CCA would be a very poor competitive substrate for the restrictive Mod5. However, this model also raises the possibility that paucity of cy-tRNA-i^**6**^A37 in higher eukaryotes reflects an IPTase type hyper-restriction that expanded to additional cy-tRNA isoacceptors. In this case, ASLs with A36-A37-A38 that could not have been modified may have undergone A37G substitution during evolution (Table 1). For example, TRIT1 might be restrictive against human cy-tRNA^**Tyr**^GUA-G37-A38 even if it had A37 because it bears other ASL determinants that negatively impact activity. In such a model, TRIT1 would modify the human mt-tRNAs-i^**6**^A37 because they do not bear negative determinants. An attempt to experimentally test a prediction of this model has not been performed. In addition, a restrictive vs. nonrestrictive type analysis had not been extended to a higher eukaryotic IPTase.

Here, we first establish that TRIT1 uses an N-terminal MTS in human cells, and that its function is required for modification of mt-tRNAs, but not cy-tRNAs, in *S*. *pombe* and *S*. *cerevisiae*. In addition, new phylogenetic and experimental analyses advance our understanding of tRNA-i^**6**^A37 identity determinants in eukaryotes. Presence of G37 rather than A in the tRNAs for UNN codons is the major determinant of the progressive paucity of cy-tRNAs-i^**6**^A37 in eukaryotes including human. A role for IPTases independent of A36-A37-A38 appears influential but limited to substrate-specific restriction against tRNA^**Trp**^CCA-A37-A38. We show that the generally nonrestrictive Mod5 and TRIT1 are restrictive against tRNA^**Trp**^CCA-A37-A38, largely dependent on the source of the tRNA, *S*. *cerevisiae* or *S*. *pombe* which are different in their ACLs only at position 32. Additional data support that C32 is part of a tRNA^**Trp**^CCA substrate-specific inhibitory circuit for Mod5 (and TRIT1). The collective data advance knowledge of how tRNA sequence and IPTase activities contribute to cy- and mt- tRNA-i^**6**^A37 profiles.

## Results

### TRIT1 has a mitochondrial targeting sequence (MTS) but is not expected to undergo cleavage

Mitochondrial targeting of human TRIT1 was predicted by the online tool MitoProt II [14] which also predicted a proteolytic cleavage site after amino acid 47 [52]. However, such cleavage would remove the conserved P-loop involved in DMAPP binding and catalysis (Fig 1, and below). We used the prediction algorithm MitoFates which was designed as an improved method for predicting cleavable N-terminal MTSs, referred to as presequences, and their cleavage sites [53]. MitoFates predicted a MTS comprised of a TOM20 (translocase of the outer mitochondrial membrane-20) receptor binding site at amino acids 5–10 (AAARAV, Fig 1) followed by an amphipathic helix at position 11–23 (PVGSGLRGLQRTL), but with the low probability of cleavage of 0.106, well below the default cutoff of 0.5. We note that the cNLS mapper [54] predicted overlapping mono- and bi-partite importin-dependent nuclear localization signals at amino acids 420–453 of TRIT1.

**Fig 2:**
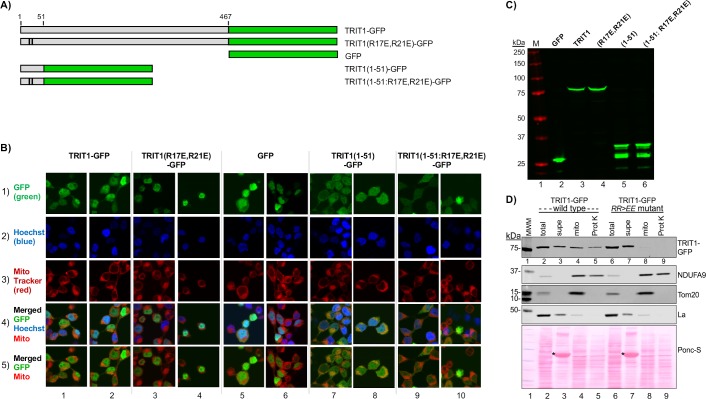
Human TRIT1 contains an N-terminal mitochondrial targeting sequence (MTS). **A)** Cartoon of the GFP-fusion constructs. Numbering represents amino acids of TRIT1; black vertical lines indicate positions of two mutated residues in the R17E/R21E constructs. **B)** Representative confocal microscopic images from HEK293 cells transfected 48 hours prior with the constructs in A and stained with Hoechst and MitoTracker. **C)** Western blot from cells transfected with constructs in A) using anti-GFP antibody (Ab). **D)** Western blot subcellular fractionation analysis. Transfected HEK293 cells were fractionated 48-hour later into supe (cytosol), mitochondria (mito) and mitochondria treated with Proteinase K (ProtK), the latter to digest proteins on the outer membrane of the mitochondria. GFP-TRIT1 WT and RR>EE mutant was detected using anti-GFP Ab. NDUFA9 is a mitochondrial inner membrane protein, La is a nuclear and cytosolic protein, TOMM20 is on the mitochondrial outer membrane. Total protein staining with Ponceau S is shown in the lower panel. Asterisks indicate BSA that was added during the mitochondrial isolation protocol (see text).

We created multiple GFP fusion constructs to test for mitochondrial localization by confocal microscopy of human embryonic kidney (HEK)-293 cells (Fig 2A and 2B). To test for MTS activity of the TRIT1 N-terminal region we fused only its first 51 amino acids to GFP, as in TRIT1(1–51)-GFP, and examined it for mitochondrial targeting. Two key point mutations, R17E, R21E, in the basic amphipathic helix component of the predicted MTS (Fig 1) were made in two contexts, the N-terminal fusion 1–51 construct, TRIT1(1–51:R17E/R21E)-GFP, and full length TRIT1(R17E/R21E)-GFP. These and other constructs were transfected into HEK293 cells. After 48 hr. the cells were exposed to MitoTracker to stain mitochondria red, fixed and treated with Hoechst to stain nuclei blue (Fig 2B). Cell lysates were examined for protein expression by western blot using anti-GFP antibody (Fig 2C). Because a significant fraction of TRIT1 localizes to nuclei, it was helpful to visualize the patterns in which the Hoechst stained nuclei were and were not included in the merged images as in rows 4 and 5 of Fig 2B respectively. Full length TRIT1-GFP was found localized to nuclei and cytosol with some evidence of mitochondrial localization reflected by overlap of GFP and MitoTracker as a stippling yellow-orange pattern in the merged panels (Fig 2B, columns 1 and 2, lower two panels). This pattern was not observed with full length TRIT1(R17E/R21E)-GFP containing the MTS mutations, which instead accumulated mostly in nuclei and was not found to localize to mitochondria (Fig 2B, columns 3 and 4). Even the least bright TRIT1-GFP expressing cells in the lower left corner of column 1, row 1 show orange stippling in column 1 row 5, whereas cells of comparable GFP brightness in column 3, row 1, show more red stippling in column 3 row 5.

Additional confocal evidence of mitochondrial localization by the TRIT1 MTS came from the TRIT1(1–51)-GFP construct. GFP alone produced a diffuse pattern with nuclei generally more intense than cytoplasm (columns 5 and 6). By contrast, TRIT1(1–51)-GFP (columns 7 and 8) was generally more intense in the cytoplasm and with distinct punctate foci that colocalized with MitoTracker in the transfected cells. Importantly, colocalized punctate foci were not observed with the MTS mutant, TRIT1(1–51:R17E/R21E)-GFP (Fig 2B, columns 9 and 10).

### Mitochondrial fractionation shows that the R17E, R21E mutations debilitate the TRIT1 MTS

We next performed subcellular fractionation on HEK293 cells after transfection of full length TRIT1-GFP and MTS mutant, TRIT1(R17E/R21E)-GFP. Western blots of total cell extracts, protein contents of their mitochondrial subfractions and supernatants, as well as of mitochondria after proteinase K-treatment, are shown in Fig 2D. TRIT1-GFP was detectable in total cell extract and supernatant (lanes 2 & 3), as well as in the contents of the mitochondrial and the proteinase K-treated mitochondria fractions (lanes 4 & 5), consistent with TRIT1 presence in the mitochondrial matrix, whereas TRIT1(R17E/R21E)-GFP was relatively lacking in the mitochondrial and proteinase K-treated mitochondria samples (lanes 8 & 9). This is consistent with localization of TRIT1 to nuclei and cytoplasm including in the mitochondrial matrix where its mt-tRNA substrates reside as previously determined by subcellular fractionation [14]. Additional analysis of the same fractions revealed the expected results for NDUFA9, an inner mitochondrial membrane protein, evident by its comparable detection in the mitochondrial and proteinase K treated mitochondria fractions in both wild-type TRIT1 and the MTS (R17E/R21E) (RR>EE) mutant TRIT1 extracts (NDUFA9, lanes 4 & 5, and 8 & 9). Likewise, the expected fractionation was also observed for Tom20, a mitochondrial outer membrane protein, in both sample sets, in the mitochondrial (Tom20, lanes 4 & 8) but not the proteinase K treated mitochondria fractions (lanes 5 & 9), and for La, an abundant soluble nuclear and cytoplasmic protein (Fig 2D, as indicated). We note that the slight mobility differences observed for the TRIT1-GFP bands in lanes 3 and 7 and their neighboring lanes is likely due to perturbation from BSA added to those samples as per the protocol (asterisks in Fig 2D bottom panel).

Our collective data support the idea that TRIT1 contains multiple trafficking elements that distribute it to nuclei, cytoplasm and inner mitochondrial matrix, similar to Mod5 [34–36, 38, 55, 56]. The MTS R17E/R21E mutations shifts distribution away from mitochondria, mostly to nuclei (Fig 2B). Importantly, as will be shown below, the MTS mutations impair i^**6**^A37 modification of mt-tRNAs specifically, without impairing modification of the nuclear-encoded cy-tRNAs by TRIT1.

### TRIT1 MTS-dependent i^6^A37 modification of mt-tRNAs in *S*. *pombe*

**Fig 3:**
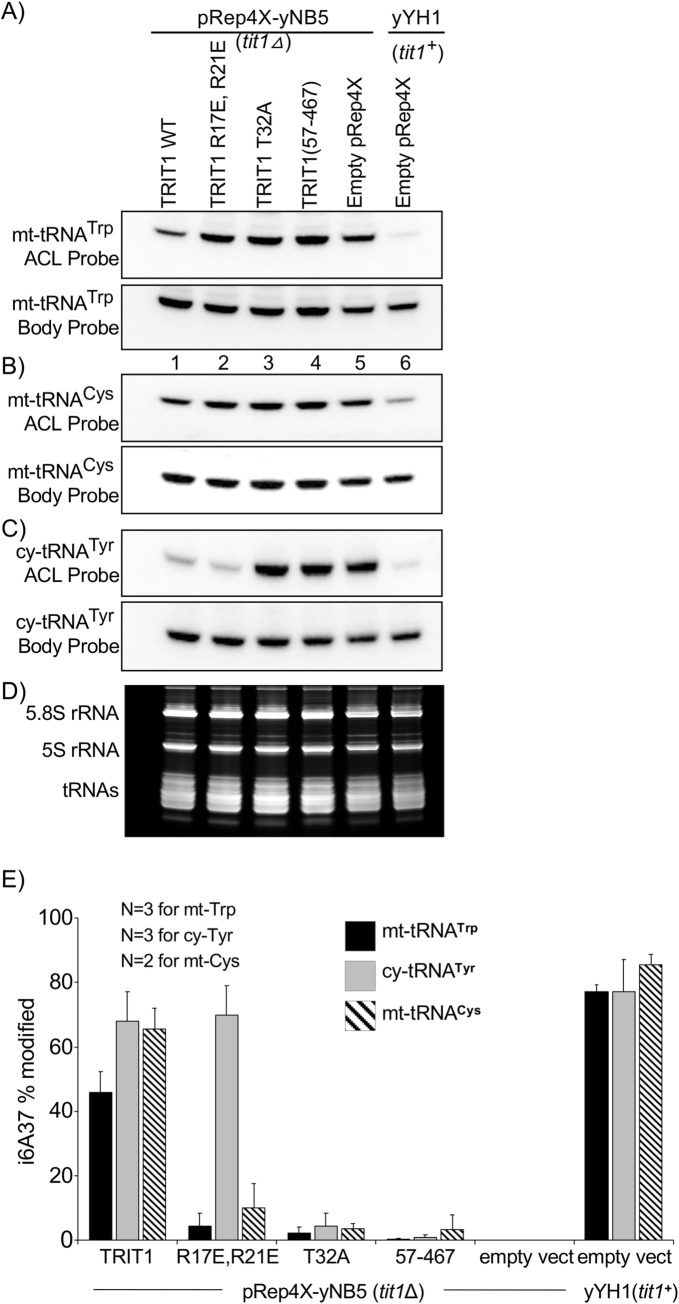
TRIT1 MTS-dependent modification of mt-tRNAs in *S*. *pombe*. **A-C)** The PHA6 northern blot assay (Positive Hybridization in Absence of i6A37) uses i^**6**^A37-sensitive ACL probes. This assay is based on loss of oligo-DNA probe hybridization to the ACL probe due to presence of the bulky i^**6**^A modification, and is calibrated for quantitation by a body probe to the T stem loop on the same tRNA. The panels show sequential probing (after stripping, Methods) for mt-tRNA^**Trp**^CCA, mt-tRNA^**Cys**^GCA, and cy-tRNA^**Tyr**^GUA with ACL and body probes as indicated; lanes are numbered below panels A. Samples in lanes 1–4 are from *tit1△* cells (yNB5) transformed with IPTases indicated above the lanes or empty vector (lane 5); lane 6 is from positive control, *tit1*^***+***^ (yYH1) cells. **D)** shows the small RNA region of the gel after staining with EtBr. **E)** Quantitation of % i^**6**^A37 modification of the tRNAs in A-C from biological duplicate, or triplicate PHA6 blots as indicated; error bars indicate standard deviation (SD).

Mitochondrial protein import pathways have been functionally conserved in eukaryotes including mammals and yeasts [57, 58]; *S*. *cerevisiae*, *S*. *pombe*, *D*. *melanogaster* and *H*. *Sapiens* share Tom20, five other TOM homologs, and twelve of the thirteen TIM (translocase of the inner mitochondrial membrane) system components [58]. Analysis of *S*. *pombe* mt-DNA by tRNAscan-SE predicts 25 mt-tRNAs (including 3 for Met), of which two, mt-tRNA^**Trp**^CCA and mt-tRNA^**Cys**^GCA contain A36-A37-A38. We examined the R17E/R21E point mutations that impaired mitochondrial localization in human cells for effects on mt-tRNA and cy-tRNA modification in *S*. *pombe* (Fig 3A–3E). For this, we used the unaltered *nmt1*^***+***^ promoter in the pRep4X expression vector as was done previously to examine mt-tRNA^**Trp**^CCA modification by Mod5 in *S*. *pombe* [6]. MOD5 uses alternative translation start sites, M1 to target the MTS-containing protein to mitochondria and cytoplasm, and M12 which produces the MTS-lacking protein that accumulates in *S*. *cerevisiae* cytoplasm and nuclei [34, 35]. Fig 3A shows results of an i^**6**^A37-sensitive PHA6 blot assay for mt-tRNA^**Trp**^CCA, mt-tRNA^**Cys**^GCA and cy-tRNA^**Tyr**^GUA. The PHA6 (Positive hybridization in Absence of i6A37) assay is based on loss of oligo-DNA probe hybridization to the ACL due to presence of i^**6**^A37, and is calibrated for quantitation by a body probe to the T stem loop on the same tRNA (Fig 3A upper and lower panels) [6, 43, 59, 60]. Here, pRep4X-TRIT1 led to ~45% i^**6**^A37 modification of mt-tRNA^**Trp**^CCA and ~65% modification of mt-tRNA^**Cys**^GCA (Fig 3A, 3B & 3E). By contrast, TRIT1(R17E/R21E) led to ~6% i^**6**^A37 modification of mt-tRNA^**Trp**^CCA and ~10% modification of mt-tRNA^**Cys**^GCA, a 7.5 and 6.5 fold reduction for these mt-tRNAs respectively, but showed high modification (~70%) of cy-tRNA^**Tyr**^GUA in the same cells, no reduction compared to TRIT1 (Fig 3A, 3C and 3E). Thus, the R17E/R21E mutations that impaired TRIT1 MTS-mediated mitochondrial localization in HEK293 cells, also impaired modification of mt-tRNA^**Trp**^CCA and mt-tRNA^**Cys**^GCA in *S*. *pombe*, while they had no effect on modification of cy-tRNA^**Tyr**^GUA (Fig 3C and 3E), the latter of which is evidence that the R17E/R21E mutations do not impair enzymatic activity. Furthermore, as the mtDNA-encoded tRNAs reside in the mitochondrial matrix and are modified in a TRIT1 MTS-dependent manner, these data provide evidence of functional localization of the TRIT1 enzyme to the mitochondrial matrix of *S*. *pombe* cells.

Substitution of invariant Thr-12 in the P-loop of Tit1 inactivates it for cy-tRNA^**Ser**^UCA TMS [43]. Tit1 T12A is homologous to T19A of *E*. *coli* IPTase which resulted in the largest decrease in *k*_***cat***_ (600-fold) of all mutations tested [47]. Indeed, the homologous T23 in Mod5 is positioned for catalysis [24]. We show here that as expected, the corresponding TRIT1-T32A mutant was inactive on both the mt- and cy- tRNA substrates (Fig 3A–3E). We also examined a variant isoform, TRIT1(57–467) [61], missing the P-loop entirely as well as D55 (see Fig 1), which in Mod5 acts as a general base for catalysis [24]. This variant represents an isoform from which translation was proposed to start at the second methionine, M57, and could complement cy-tRNA^**Tyr**^UUA TMS in *S*. *cerevisiae* [61]. Our TRIT1(57–467) construct was inactive on the mt-tRNA and cy-tRNA substrates (Fig 3A–3E).

### TRIT1 MTS-dependent i^6^A37 modification of mt-tRNAs in *S*. *cerevisiae*

**Fig 4:**
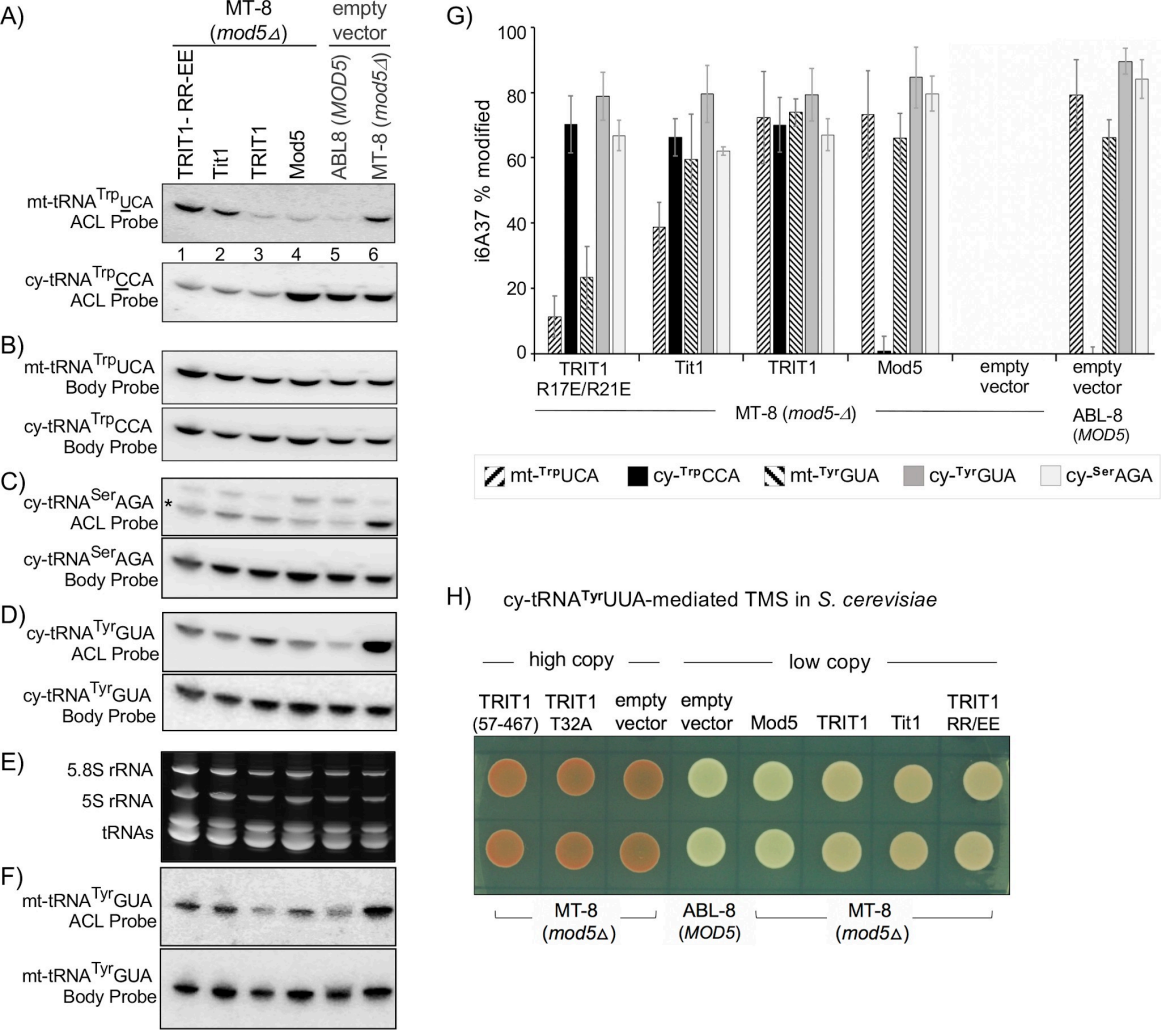
TRIT1 MTS-dependent modification of mt-tRNAs in *S*. *cerevisiae*. **A)** A PHA6 i^**6**^A37-sensitive northern blot after transformation of the strains indicated above the lanes, showing sequential probing for mt-tRNA^**Trp**^UCA and cy-tRNA^**Trp**^CCA with the respective ACL-specific probes. MT-8 is *mod5Δ* and ABL-8 is a *MOD5* positive control. **B)** The same blot showing reprobing (after stripping, Methods) with the body probes for mt-tRNA^**Trp**^UCA and cy-tRNA^**Trp**^CCA as indicated. **C)** The same blot showing ACL and body probings for cy-tRNA^**Ser**^AGA; asterisk indicates mature tRNA species, upper band is likely a cy-tRNA^**Ser**^AGA gene-specific precursor. **D)** The same blot showing ACL and body probings for cy-tRNA^**Tyr**^GUA with ACL and body probes as indicated. **E)** The small RNA region of the gel after staining with EtBr. **F)** Shows the ACL and body probe results for mt-tRNA^**Tyr**^GUA from a different blot. **G)** Quantitation of % i^**6**^A37 modification of the tRNAs in A-D, F from biological duplicate, or triplicate PHA6 blots as indicated; error bars indicate standard deviation (SD). **H)** Cy-tRNA^**Tyr**^UUA-mediated TMS in *S*. *cerevisiae* MT-8 (*mod5Δ*) transformed with empty vector or the IPTases indicated.

We examined TRIT1 and other IPTases in the *mod5Δ*, *S*. *cerevisiae* strain, MT-8 [34]; ABL8 is a MOD5 replete strain. PHA6 blot analysis for multiple tRNAs are shown in Fig 4, including quantifications in replicate. Fig 4A shows the ACL probings for mt-tRNA^**Trp**^UCA and cy-tRNA^**Trp**^CCA, with the body probings for these tRNAs in Fig 4B. Results for cy-tRNA^**Ser**^AGA, cy-tRNA^**Tyr**^GUA and mt-tRNA^**Tyr**^GUA are shown in Fig 4C–4G. TRIT1 modified the three cy-tRNAs and both mt-tRNAs in *S*. *cerevisiae* whereas TRIT1-R17E/R21E was deficient only for the mt-tRNAs. TRIT1-R17E/R21E-mediated modification of cy-tRNAs but not mt-tRNAs supports the conclusion that the TRIT1 MTS is functional for delivery of TRIT1 IPTase activity to the inner mitochondrial matrix in *S*. *cerevisiae*.

Three additional points are relevant. First, substrate-specific restriction against cy-tRNA^**Trp**^CCA-A37-A38 by Mod5 relative to the efficient modification of the mt-tRNA^**Trp**^UCA-A37-A38, mt-tRNA^**Tyr**^GUA-A37-A38, cy-tRNA^**Ser**^AGA, and cy-tRNA^**Tyr**^GUA, was demonstrated for the endogenous and ectopic Mod5 enzyme (compare both panels of Fig 4A, and upper panels of C, D, F, and panel G). Second, TRIT1 modified *S*. *cerevisiae* cy-tRNA^**Trp**^CCA-A37-A38 with efficiency similar to Tit1 and with efficiency similar to Mod5 for the other tRNAs (Fig 4G). Third, Tit1 showed somewhat low activity on the mt-tRNAs (Fig 4G). We suspect this may reflect a mitochondrial import mechanism that isn't fully efficient for Tit1 in *S*. *cerevisiae*, consistent with the alignment in Fig 1 although other explanations are possible.

We also examined the IPTases for cy-tRNA^**Tyr**^UUA-mediated TMS in *S*. *cerevisiae* which is dependent on efficient i^**6**^A37 modification. This revealed that while TRIT1, Tit1 and TRT1-R17E/R21E (TRIT1-RR/EE) were active, TRIT1-T32A and TRIT1(57–467) were inactive (Fig 4H). We note that for TMS, Mod5, Tit1, TRIT1 and TRIT1-TRT1-R17E/R21E were expressed from a low copy plasmid whereas TRT1-T32A and TRT1(57–467) were from a high copy plasmid (Fig 4H).

### Complementation of cy-tRNA^Ser^UCA-mediated suppression in *S*. *pombe*

**Fig 5:**
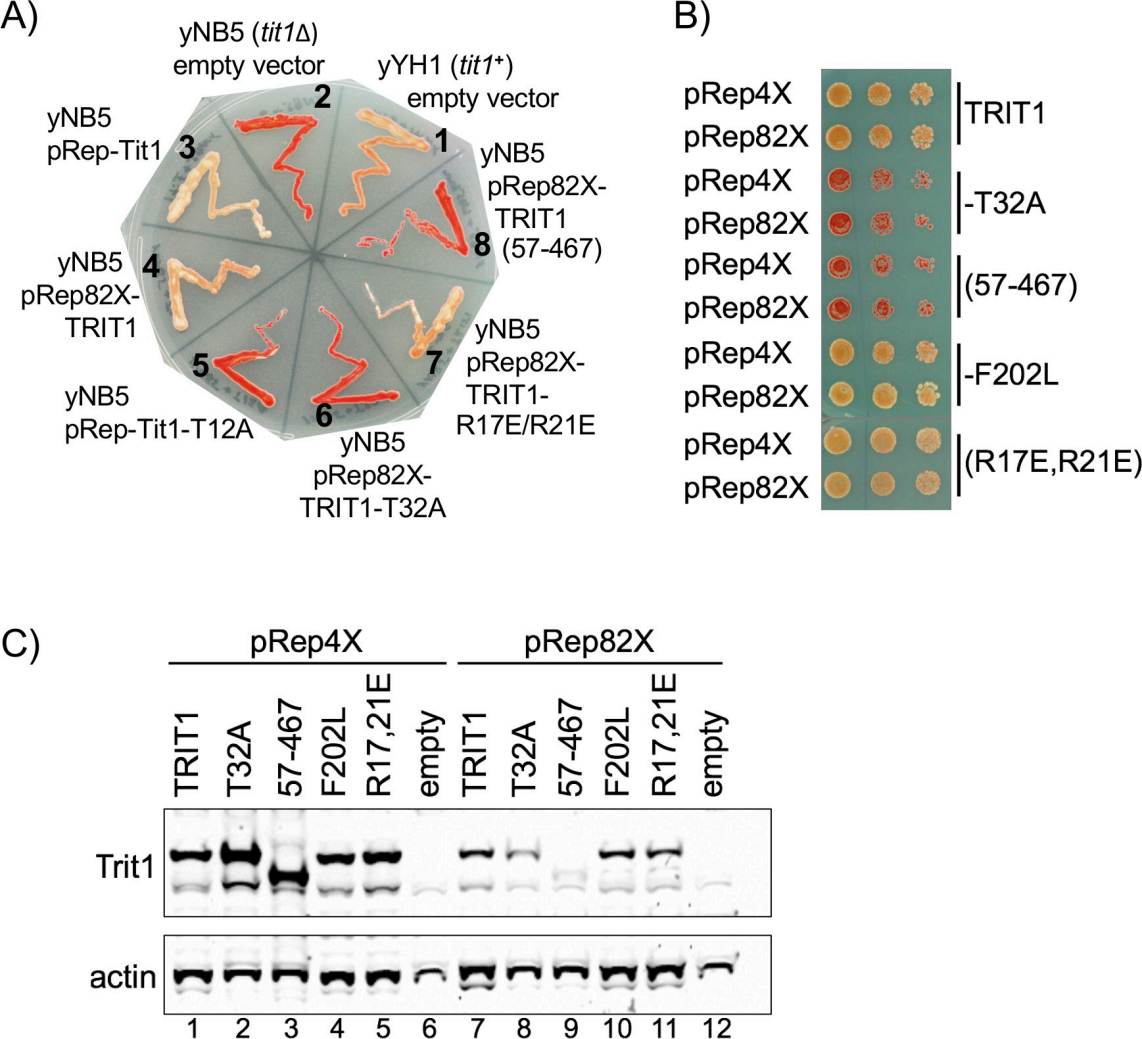
Determinants of TRIT1 critical for cy-tRNA^Ser^UCA-mediated TMS in *S*. *pombe*. **A)** Cy-tRNA^**Ser**^UCA-mediated TMS assay in *S*. *pombe* yNB5 (*tit1Δ*) transformed with the empty plasmid pRep82X (sector 2) or plasmids expressing the proteins indicated (sectors 3–8), and the *tit1*^***+***^ control strain, yYH1 (sector 1). **B)** TMS assay as in A but displayed as dilution spot assay, for plasmids expressing TRIT1 and variants from pRep82X and from the stronger *nmt1*^***+***^ promoter (pRep4X) as indicated on the left. **C)** Western blot of extracts of cells in B detected with anti-TRIT1 (top panel), and anti-actin antibody; lanes numbers are indicated below actin panel.

The monitoring of codon-specific insertion of an amino acid in the β-galactosidase active site was used to show that i^**6**^A37 increases tRNA decoding activity of the two different tRNAs tested by 3 to 4-fold in *S*. *pombe*, consistent with i^**6**^A37 leading to general increased tRNA affinity for ribosome decoding [60]. TMS makes use of a codon-specific reporter to examine TRIT1 mutants for modification activity of cy-tRNA^**Ser**^UCA in *S*. *pombe* deleted of *tit1*^***+***^ (yNB5 *tit1Δ*, Fig 5A). Cy-tRNA^**Ser**^UCA is dependent on efficient i^**6**^A37 modification for serine insertion at codon 215 of *ade6-704* mRNA [62] with consequent decrease in cellular red pigment during growth in limiting adenine [33]. The pathogenic *TRIT1-R323Q* variant exhibits substantially reduced modification activity relative to TRIT wild type in the *S*. *pombe* TMS assay using the pRep82X expression vector [14]. In this assay, the positive control strain yYH1 (*tit1*^***+***^) is pink (moderately suppressed) whereas yNB5(*tit1Δ*) transformed with empty vector is unsuppressed, red (Fig 5A, sectors 1, 2). Transformation of yNB5 with pRep-Tit1 under control of the *tit1*^***+***^ promoter, and pRep82X-TRIT1, led to suppression (sectors 3, 4). The catalytic mutants, pRep-Tit1-T12A and pRep82X-TRIT1-T32A, were inactive, as was pRep82X-TRIT1(57–467) (sectors 5, 6, 8), consistent with the PHA6 results in Fig 3.

Importantly, pRep82X-TRIT1(R17E/R21E) was active for TMS (Fig 5A, sector 7) comparable to pRep82X-TRIT1. This provided additional evidence that the R17E/R21E mutations that impair mitochondrial targeting do not impair enzymatic functional activity for the cy-tRNA.

### Moderate over-expression does not rescue inactive TRIT1 alleles for cy-tRNA^Ser^UCA TMS

Human TRIT1 was identified as a candidate tumor suppressor [61]. An allele that encodes TRIT1-F202L was later found to be associated with modulation of lung cancer survival [63] (also see [64], [65]) and was reported to be active for cy-tRNA^**Tyr**^UAA-TMS in *S*. *cerevisiae* using a high level expression plasmid system [61]. Therefore, we wanted to examine TMS using both a weakened and a stronger promoter. The pRep4X promoter fused to chloramphenicol acetyltransferase (CAT) or *LacZ*, showed 45–75 fold more output than pRep82X, the latter of which was created by mutating the TATA box of the natural *nmt1*^***+***^ promoter [66, 67]. pRep4X-TRIT1(57–467) indeed produced more TRIT1(57–467) than did pRep82X-TRIT1(57–467) (Fig 5C), although without a hint of increased TMS activity relative to pRep82X-TRIT1(57–467) (Fig 5B). TRIT1-T32A also remained inactive in pRep4X (Fig 5B and 5C). TRIT1-F202L was as active as TRIT1 and not limited by expression levels, and this was also true for TRIT1-(R17E/R21E) (Fig 5B and 5C).

### TRIT1 exhibits substrate-specific restriction against modification of *S*. *pombe* cy-tRNA^Trp^CCA

**Fig 6:**
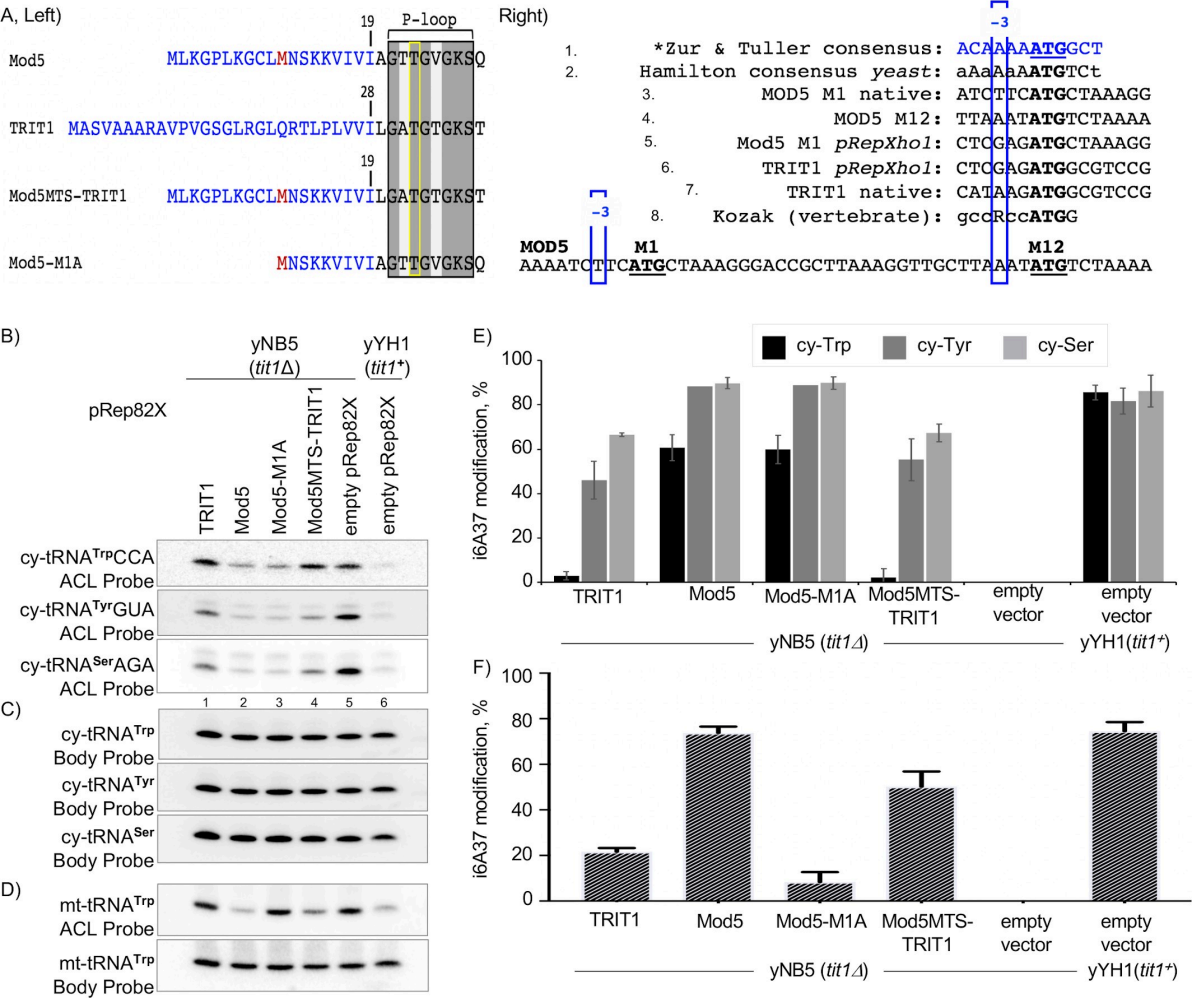
TRIT1 exhibits substrate-specific restriction against cy-tRNA^Trp^CCA in *S*. *pombe*. **A)** Schematics showing constructs used for analysis of tRNA i^**6**^A37 modification in *S*. *pombe*. Left: amino acid sequence representation of N-termini only, similar to Fig 1; the invariant Thr demarcated by yellow rectangle. The second, alternative translation initiation sites, where present are shown in red. Right: translation initiation contexts of the various constructs used. Lines 1, 2 and 8 represent translation start site consensuses as indicated [101–103], the former of which was derived for *S*. *cerevisiae* but applies to other yeasts including *S*. *pombe*, at the -3 position. **B)** The i^**6**^A37-sensitive PHA6 northern blot assay for three tRNAs from *S*. *pombe* expressing IPTases from pRep82X plasmids indicated above the lanes. The blot was sequentially probed with the i^**6**^A37-sensitive ACL probes to the cy-tRNAs indicated to the left of the panels. **C)** The body probes for each of the tRNAs in B. **D)** The same blot as in B-C, probed for mt-tRNA^**Trp**^CCA using the ACL and body probes indicated. **E)** Quantitation of % i^**6**^A37 modification of the cy-tRNAs from biological replicate PHA6 northern blots as in panels B-C; n = 5 for cy-tRNA^**Trp**^CCA and all others except n = 1 for cy-tRNA^**Tyr**^ in Mod5 and Mod5M1A. **F)** Quantitation; % i^**6**^A37 modification of mt-tRNA^**Trp**^CCA from biological duplicate PHA6 northern blots; error bars indicate standard deviation (SD).

We note that while comparison of different IPTases in a heterologous '*in vivo*' system is not physiological, it provides an experimental model with which to gauge activity of an ectopic IPTase for a particular tRNA relative to other tRNA substrates in the cells and compare the overall tRNA substrate activity pattern to that of a different ectopic IPTase in the same system. We did that for Fig 3 by using *S*. *cerevisiae* as the heterologous system and in Fig 6 using *S*. *pombe* as the heterologous system. Unexpectedly, the cy-tRNA^**Trp**^CCA-A37-A38 in *S*. *pombe* was a very poor substrate of TRIT1 as compared to cy-tRNA^**Ser**^AGA and cy-tRNA^**Tyr**^GUA, and Tit1 (Fig 6). Unexpected because TRIT1 efficiently modified the *S*. *cerevisiae* cy-tRNA^**Trp**^CCA-A37-A38 in *S*. *cerevisiae*. Similarly, it was unexpected that Mod5 would modify the *S*. *pombe* cy-tRNA^**Trp**^CCA-A37-A38 to as high as 60% (Fig 6E) based on its restriction against *S*. *cerevisiae* cy-tRNA^**Trp**^CCA-A37-A38.

Effects of increasing TRIT1 levels on tRNA modification were also examined. Two additional IPTase constructs were included in this analysis, Mod5-M1A and Mod5MTS-TRIT1, the latter of which was created by replacing the first twenty-eight codons of TRIT1 with the first nineteen codons of MOD5 which includes sequences in its MTS and the second translation initiation site at M12, as schematized in the left and right panels of Fig 6A. In addition to usefulness for assessing mt-tRNA modification, the MOD5 leader sequence also produces increased levels of cytoplasmic isoforms of the IPTases because of the known high favorability of the translation initiation context of the second methionine, M12 relative to MOD5 M1 [68] and TRIT1 M1 (Fig 6A, S1A–S1D Fig). Mod5 and Mod5-M1A were only modestly deficient for cy-tRNA^**Trp**^CCA modification, whereas TRIT1 and Mod5MTS-TRIT1 were severely deficient for cy-tRNA^**Trp**^CCA modification relative to cy-tRNAs ^**Tyr**^GUA and ^**Ser**^AGA (Fig 6B, 6C & 6E). Hypomodification of cy-tRNA^**Trp**^CCA is reflected by strong hybridization signals in lanes 1 and 4 relative to lanes 2 and 6 of the PHA6 ACL probe blots in Fig 6B, upper panel, and by the biological replicate data in Fig 6E. The data indicate that while Mod5 sees the *S*. *pombe* cy-tRNA^**Trp**^CCA as the least preferred of the cy-tRNA substrates, this is modest compared to the more severe substrate-specific restriction against this tRNA by TRIT1. The cy-tRNA^**Trp**^CCA is efficiently modified by Tit1 consistent with previous data [43].

We confirmed that Mod5-M1A, which initiates at M12 and therefore lacks a MTS [34, 35], is deficient for mt-tRNA^**Trp**^CCA modification [6] (Fig 6D and 6F). Notably, pRep82X-TRIT1 reproducibly modified mt-tRNA^**Trp**^CCA to 20% in the same cells in which it modified cy-tRNA^**Trp**^CCA to only 5% (Fig 6E). Mod5MTS-TRIT1 exhibited 50% modification of mt-tRNA^**Trp**^CCA, ~2.5-fold higher than TRIT1 (Fig 6F). Analysis of protein expression levels indicate that levels of cytoplasmic isoform of TRIT1 accumulate to >10-fold higher from pRep82X-Mod5MTS-TRIT1 relative to pRep82X-TRIT1 (S1C and S1D Fig). However, this had little if any effect on the modification efficiency of *S*. *pombe* cy-tRNA^**Trp**^CCA (Fig 6E). *S*. *pombe* cy-tRNA^**Trp**^CCA modification can be increased by increasing TRIT1 protein levels, but this requires even higher expression, from pRep4X-Mod5MTS-TRIT1 (S1G and S1H Fig).

### Position 32 influences tRNA^Trp^CCA substrate-specific restriction by Mod5 and TRIT1 IPTases

Our data indicate that Mod5 and TRIT1 exhibit differential restriction against the cy-tRNAs^Trp^CCA of *S*. *cerevisiae* and *S*. *pomb*e which differ at C32 vs. U32 as well as other positions more distant to their ACLs (S2 Fig). Given the modification circuit relationship between position 32 and i^**6**^A37 among tRNAs^**Ser**^ [12, 13] and other potential effects of position 32 on ACL structure (and function [69, 70]), we suspected position 32 as a possible determinant of the differential activities of TRIT1 and Mod5 on the *S*. *cerevisiae* and *S*. *pombe* cy-tRNAs^**Trp**^CCA.

**Fig 7:**
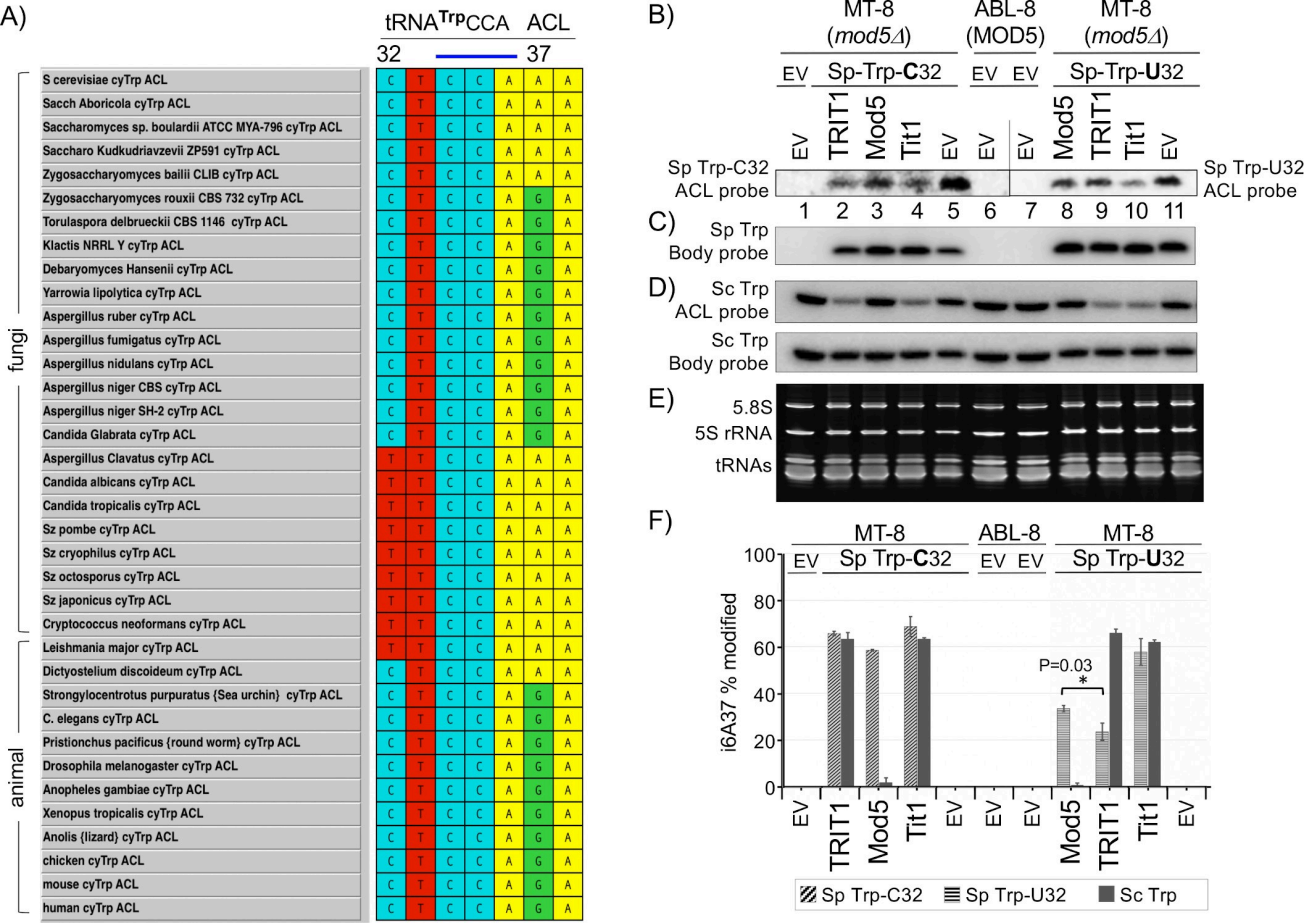
Position 32 as a determinant of differential IPTase activity for cy-tRNA^Trp^CCA in eukaryotes. **A)** Sequence alignment of the cy-tRNA^Trp^CCA ACLs in the eukaryotes indicated; the 32 and 37 positions are numbered above; a horizontal blue bar indicates the AC. **B)** Results of PHA6 i^**6**^A37-sensitive PHA6 northern blot after expressing a *S*. *pombe* cy-tRNA^**Trp**^CCA gene in MT-8 (*mod5Δ*) *S*. *cerevisiae* containing the native U32 (right panel) or this position mutated to C32 (left panel), or empty vector (EV) as indicated above the lanes, in the presence the IPTases or empty vector (EV) also indicated above the lanes; ABL-8 is a *MOD5* positive strain. The right and left panel blots show separate hybridizations with ACL probes. Note that the order of the IPTases in the left and right panels differs, and carries through to panel F. **C)** The body probe result for *S*. *pombe* cy-tRNA^**Trp**^CCA. **D)** ACL probe (upper) and body probe (lower) results for endogenous *S*. *cerevisiae* cy-tRNA^**Trp**^CCA as indicated. **E)** shows the EtBr-stained gel from which the blot was made. **F)** Quantification of % i^**6**^A37 modification of the three different cy-tRNAs^**Trp**^CCA as indicated; error bars indicate standard deviation (SD) from biological duplicates. P value was obtained by the paired t-test.

To attempt to gain broader perspective we collected tRNA gene sequences encoding the ACLs of cy-tRNAs^**Trp**^CCA in representative related yeasts as well as animal cells available in an appropriate database [71] and subjected them to multiple sequence alignment (Fig 7A). Three patterns emerged. The related *Saccharomyces* and *Zygoaccharyomyces bailii* species, at the top of the alignment, revealed a C32-A37 pattern different from the other fungi. The remaining nineteen fungal species exhibited two different covariations; eight with T32-G37 and eleven with C32-A37 (Fig 7A). The C32-G37 pattern was predominant in the animal cell species, with the exceptions being the primitive *Leishmania* and *Dictyostelium*. For comparison, ACL alignments of cy-tRNA^**Cys**^GCA was also done which revealed a less clear modal 32–37 covariation among the fungi than for cy-tRNAs^**Trp**^CCA (S3 Fig). For all fungal cy-tRNAs^**Tyr**^GUA examined, the C32-A37 pattern was set, whereas the C32-G37 pattern was set for all eukaryotes examined (S3 Fig). The data are consistent with the idea that potential for i^**6**^A37 modification of cy-tRNAs^**Trp**^CCA was relatively variable in yeasts due to A37 vs. G37 identity and became progressively less variable and then fixed by G37 identity in higher eukaryotes.

The observed differences in relative modification of *S*. *cerevisiae* and *S*. *pombe* cy-tRNAs^**Trp**^CCA by TRIT1 and Mod5 in the two yeast systems might be due to differences in the tRNA sequences themselves, posttranslational modifications of the IPTases, or subcellular distributions. Considering the wide range of functional transferability of IPTases from multiple distant phylogenetic groups to another (Introduction), we suspected that a basis of the modification differences may be in the cy-tRNAs^**Trp**^CCA themselves. We set out to look into this by examining IPTase activity for the *S*. *pombe* cy-tRNA^**Trp**^CCA carrying its native encoded U32 or with this position mutated to C32, expressed in *mod5Δ* MT-8 *S*. *cerevisiae*. We chose this approach because *S*. *pombe* tRNA genes can be readily expressed in *S*. *cerevisiae*, but not vice versa [72], and because this tRNA can be distinguished from the *S*. *cerevisiae* cy-tRNA^**Trp**^CCA [43]. A PHA6 ACL probed blot for the *S*. *pombe* cy-tRNA^**Trp**^CCA with U32 and C32 is shown in the Right and Left panels of Fig 7B, and the body probe blot is shown in Fig 7C. The ACL probe for the endogenous *S*. *cerevisiae* cy-tRNA^**Trp**^CCA is shown in Fig 7D, upper panel, with the body probe in the lower panel. **Fig** 7E shows the lower part of the gel from which the blot was made. Panel F shows quantitation of the PHA6 data for all three tRNAs. The PHA6 analysis of the *S*. *pombe* cy-tRNA^**Trp**^CCA with U32 and C32 was consistent with Figs 4 and 6 in so far as TRIT1 was less active than Mod5 on the cy-tRNA^**Trp**^CCA with its native U32 and more active than Mod5 on cy-tRNA^**Trp**^CCA substituted with C32 (Fig 7B and 7C) which better matches the *S*. *cerevisiae* cy-tRNA^**Trp**^CCA ACL. The data showed that substrate-specific restriction by TRIT1 against *S*. *pombe* cy-tRNA^**Trp**^CCA could be demonstrated in *S*. *cerevisiae*, while maintaining efficient modification of endogenous *S*. *cerevisiae* cy-tRNA^**Trp**^CCA (Fig 7F), with a trend of general relative activities similar to observed for TRIT1 in the two yeasts. Moreover, the single point mutation, U32C, converted *S*. *pombe* cy-tRNA^**Trp**^CCA from a poor to better substrate of TRIT1, while Tit1 modified both substrates to comparable efficiencies (Fig 7B and 7F).

However, this experiment also produced unexpected results that raised a new hypothesis. Mod5 was more active on the C32 tRNA^**Trp**^ than expected, which bears an encoded ACL that matchs that of the endogenous *S*. *cerevisiae* cy-tRNA^**Trp**^CCA, while it remained unable to modify the latter (Fig 7D and 7F). This suggests that another difference(s) in the cy-tRNAs^**Trp**^CCA of the two yeasts may be responsible, for example, G26 vs. U26 respectively (see S2 Fig).

### Evidence against a hyper-restriction model for TRIT1

**Fig 8:**
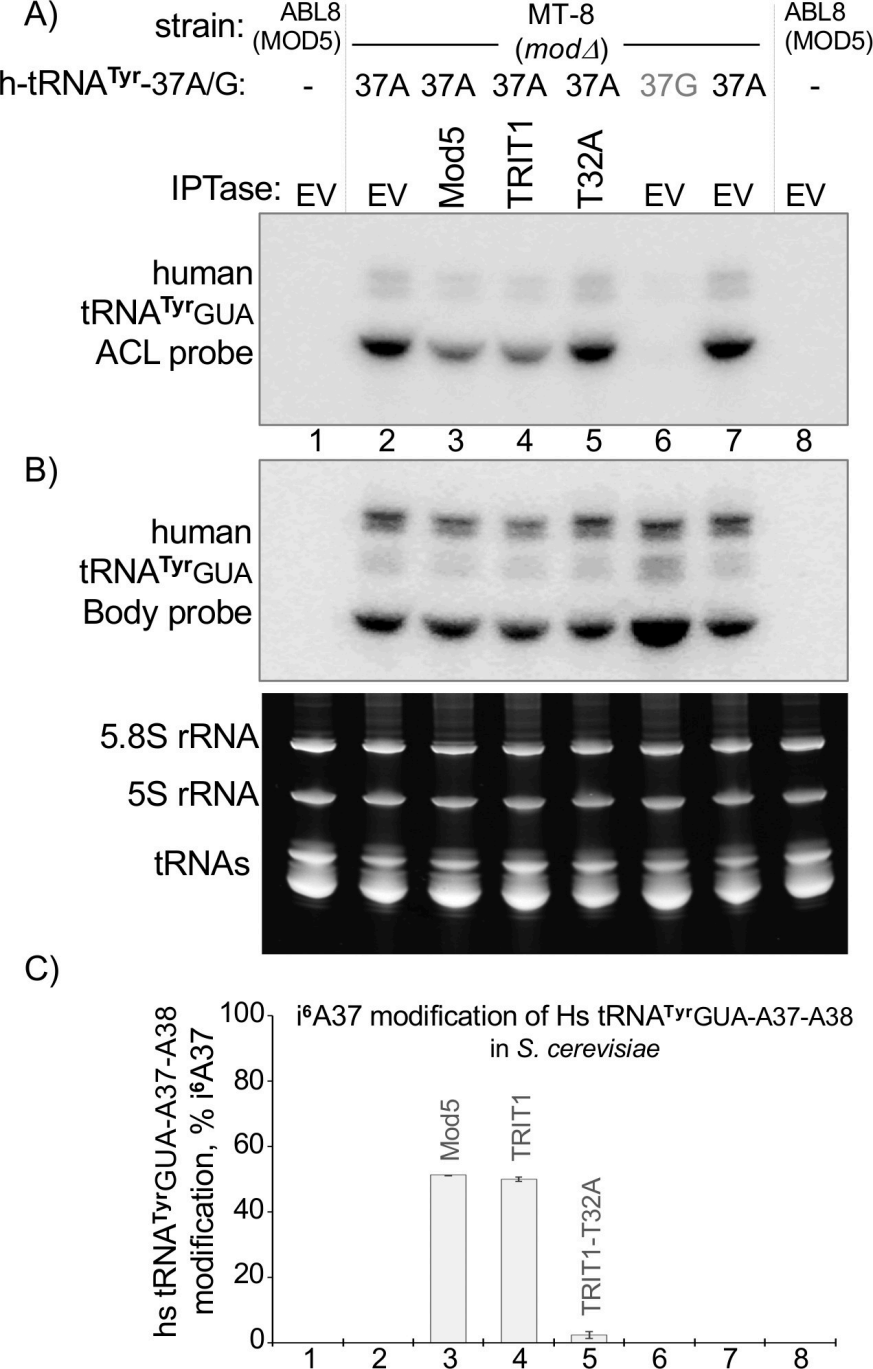
Evidence against a hyper-restriction model for TRIT1. **A)** Results of PHA6 i^**6**^A37-sensitive PHA6 northern blot assay after expressing a human nuclear gene for tRNA^**Tyr**^GUA-G37-A38 with a G37A point mutant in *mod5Δ* MT-8 *S*. *cerevisiae* cells (lanes 2–5, 7) and with native G37 (lane 6), in the presence of empty vector (EV) or the IPTases indicated above the lanes (T32A = TRIT1-T32A); lanes 1 and 8 show ABL-8, the MOD5 positive strain. **B)** Body probe of the blot in A (upper panel), and EtBr stained gel (lower panel). **C)** Quantitation of % i^**6**^A37 modification of the cy-tRNA^**Tyr**^GUA from biological duplicate PHA6 northern blots as in panels A-B; error bars indicate standard deviation (SD).

Finally, we designed an experiment to address if a hypothetical hyper-restriction IPTase model may apply to TRIT1 or if the alternative simple model of A36-A37-A38 as the principal positive determinant of activity applies. As noted in the Introduction, the hyper-restriction model would predict that TRIT1 would not modify human cy-tRNA^**Tyr**^GUA-G37-A38 even if it had A37 because other negative determinants would prevail. Whereas in the simple model, cy-tRNA^**Tyr**^GUA-A37-A38 would be a ready substrate of TRIT1. We therefore made a point mutation, G37A, in a human cy-tRNA^**Tyr**^GUA gene and examined it in MT-8 cells. This converted the tRNA^**Tyr**^GUA into a substrate that was modified with comparable efficiency by both enzymes (Fig 8). This was as expected of the simple model of A36-A37-A38 recognition in substrates that do not have a YYA anticodon.

## Discussion

In this paper, two related topics concerning i^**6**^A37 modification of cytoplasmic and mitochondrial tRNAs were advanced. First, a newly predicted MTS in the amino-terminal sequence of TRIT1 comprised of a TOM20 receptor binding site followed by an amphipathic helix was shown to be functional by the mutation of two key basic residues in the amphipathic helix. The mutations disabled targeting of TRIT1-GFP to the inner mitochondria of human cells, and impaired TRIT1-mediated i^**6**^A37 modification of mt-tRNAs in *S*. *pombe* and *S*. *cerevisiae* without affecting i^**6**^A37 modification of cy-tRNAs in the cells. Use of these yeast *in vivo* systems was critical for demonstrating function of the TRIT1 MTS in mt-tRNA modification. Understanding TRIT1 mitochondrial function is important because human TRIT1-deficiency leads to i^**6**^A37-hypomodification of mt-tRNAs and consequent impaired translation of mt-mRNAs that encode respiratory complex components [14].

That TRIT1 can access and modify cy- and mt- tRNAs in two evolutionarily diverged yeasts suggests that no specialized posttranslational modification of TRIT1 is required for function, or if so, it is highly conserved [73]. A model of IPTase action of direct recognition of the tRNAs as observed in cocrystal structures is consistent with this and the ability of phylogenetically divergent species' IPTases to complement tRNA-mediated TMS in yeast (Introduction).

This study also advanced insight into determinants of tRNA-i^**6**^A37 identity variation. It should be noted that tRNA-i^**6**^A37 variation in Table 1 is in contrast to that for threonylcarbamoyl-A37 (t^**6**^A37) which occurs nearly without exception on all tRNAs for ANN codons in all species [74, 75, reviewed in 76].

Although biological relevance of tRNA-i^**6**^A37 variation is yet unknown, that i^**6**^A37 is a functionally important modification is clear. I^**6**^A37 tRNA hypomodification that results from pathogenic mutations in TRIT1 causes debilitating childhood neurodevelopmental disorders [14, 15]. Deletion of TRIT1 is embryonic lethal in mice [77] and RNAi-mediated IPTase knockdown in silkworms leads to death [28]. Studies in *S*. *pombe* support the idea that changes in i^**6**^A37 levels can affect translation of specific mRNAs rich in i^**6**^A37-cognate codons [6, 60]. Because i^**6**^A37 formation is linked to basal metabolism, it is plausible that tRNA-i^**6**^A37 levels could vary under physiologic conditions and regulate the translation of specific mRNAs [78, see 79]. As i^**6**^A37 is a unique modification that increases tRNA affinity for the ribosome and specific activity for decoding (Introduction), it may serve its translation systems distinctively and plastically [80]. Full appreciation of i^**6**^A37 function will require better understanding of individual IPTase-tRNA systems. With the R17E,R21E mutations, researchers can attempt to specifically rescue cy-tRNA i^**6**^A37 modification in patient-derived cells while maintaining mt-tRNA hypomodification to begin to dissect the potential contribution of the cy-tRNAs to the phenotype.

This study represents comparative analyses of three IPTases and multiple tRNA substrates in two heterologous '*in vivo*' model organisms, *S*. *pombe* and *S*. *cerevisiae* deleted of their endogenous IPTases. Quantitative modification analysis used the PHA6 assay, i^**6**^A37-sensitive northern blot probing of more than ten individual tRNAs followed by tRNA-specific internal calibration for each tRNA species, five endogenous substrates in *S*. *cerevisiae*, four endogenous substrates in *S*. *pombe*, and multiple heterologous tRNAs. We showed for the first time, the large quantitative extent to which cy-tRNA^**Trp**^CCA is a poor substrate of Mod5 (endogenous and ectopic) in *S*. *cerevisiae*, while other substrates, including mt-tRNA^**Trp**^UCA, are efficiently modified in the same cells (Fig 4A–4G). IPTase activities were also examined in *S*. *pombe* (Fig 6). A conclusion is that the three divergent IPTases exhibit largely unrestrained i^**6**^A37 modification activity on a range of tRNAs-A36-A37-A38 with the exception of tRNA^**Trp**^CCA which is a least favored substrate for Mod5 and TRIT1, albeit conditional on position 32, whereas Tit1 does not discriminate against this type of substrate. Thus, although restrictive type IPTase discrimination against recognition of certain anticodons does exists in some eukaryotes, this appears to have been limited to cy-tRNA^**Trp**^CCA. This discriminatory feature was retained by TRIT1 even though its cy-tRNA^**Trp**^CCA contains G37 and is not a substrate for i^**6**^A modification. Data in Fig 7 suggest that elements beyond the ACL of *S*. *cerevisiae* cy-tRNA^**Trp**^CCA-A37-A38 contribute to the remarkable extent to which this tRNA is a specifically poor substrate of its cognate IPTase (Fig 4A and 4G).

### The N-terminal MTS of TRIT1

The mechanisms by which Mod5 localizes to mitochondria, nuclei and cytoplasm in *S*. *cerevisiae* have been well investigated [34–36, 68, 81, 82]. Mod5 and GRO-1 of the round worm, *C*. *elegans* use alternative translation initiation to target to mitochondria or cytoplasm [34, 35, 44]. Our data indicate that TRIT1 uses a single translation start site to produce mitochondrial, cytoplasmic and nuclear protein. Use of alternative translation initiation start sites is attractive because it could theoretically provide opportunity for regulation of the relative amount of the isoforms. The second methionine of the conserved metazoan IPTase is M57 of human TRIT1, part of a DSMQ motif involved in A37 recognition (Fig 1). A cDNA representing TRIT1(57–467) was isolated from human lung cDNA (referred to as Tr1 in ref [61]). This variant was found to be inactive for TMS in *S*. *pombe* and *S*. *cerevisiae* when compared to full length TRIT1 (Figs 4 and 5). It should be noted that although TRIT1(57–467)/Tr1 appeared active for TMS relative to other variants Tr2-8, it was not compared to full length TRIT1 [61] as was done here. Based on data here including TRIT1-T32A, we suggest that TRIT1 variant activities should be compared to TRIT1.

Because mt-tRNA i^**6**^A37 modification is critical to TRIT1 function we wanted to examine its mitochondrial import mechanism. As noted, MitoProt II predicted post-import cleavage at position 47, yet such a protein would lack a P-loop and the invariant T32 whose homologous mutations in TRIT1, Tit1-T12A, and MiaA inactivated them. MitoFates [53] is a newer and improved algorithm that predicted a new N-terminal MTS for TRIT1 but with low probability of cleavage. Prior subcellular fractionation showed TRIT1 localizes to the mitochondrial matrix [14]. Our data confirmed and extended this by point mutagenesis of two residues of the MTS which debilitated localization in human cells as well as i^**6**^A37 modification of mt-tRNAs, but not cy-tRNAs, in *S*. *pombe* and *S*. *cerevisiae*. In summary, our data do not support a model in which translation initiation at the second methionine, M57, nor cleavage after mitochondrial import at position 47, can produce efficient tRNA i6A37 modification activity.

Interestingly, it was concluded that the N-terminal 11 amino acids of Mod5 is required as part of a longer MTS that is probably not cleaved after import [34, 35]. This is supported by MitoFates analysis that predicts positively charged amphiphilicity in the Mod5 N-terminal region but with a very low probability of cleavage, 0.026. The earlier studies also indicate that Mod5 MTS is inefficient at import, consistent with a functional cytoplasmic pool of N-terminal extended protein [34]. Our data with Mod5MTS-M12A-TRIT1 are consistent with this as it retained significant i^**6**^A37 modification of cy-tRNAs. Perhaps related, biochemical and subcellular fractionation suggest a similar feature of TRIT1 [14]. Yarham *et al*. could not exclude the possibility that a significant fraction of cytoplasmic TRIT1 may be associated with the outer mitochondrial membrane [14]. This is consistent with microscopy that showed a substantial amount of the total cytoplasmic TRIT1-GFP and TRIT1(1–51)-GFP appeared to be mitochondria-associated (Fig 2B).

Future experiments may address the possibility that TRIT1 and Mod5 localize on the outer mitochondrial surface. Such localization has recently been noted for other tRNA ACL modification enzymes as well as processing factors for tRNA and other RNAs in yeast and metazoa [reviewed in 2]. Specifically, the enzyme responsible for formation of cyclic t^**6**^A37 [74] localizes to S. *cerevisiae* mitochondrial surface [see 2]. Similar IPTase localization might augment tRNA i^**6**^A37 formation via ready access to DMAPP, a product of the HMG-CoA reductase pathway, as it exits mitochondria, and enhance the link between i^**6**^A and basal metabolism [78, see 79].

### Different TRIT1 modification efficiencies for cy- vs. mt- tRNA^Trp^CCA-A37-A38 in *S*. *pombe*

At different TRIT1 expression levels, mt-tRNA^**Trp**^CCA-A37-A38 was modified more efficiently than cy-tRNA^**Trp**^CCA-A37-A38 (Fig 6, S1F and S1G Fig). Although the data support that the differences are significant yet do not provide mechanistic resolution, some points can nonetheless be considered. While the encoded ACL nucleotides of these cy- and mt- tRNAs^**Trp**^ are identical (S1G Fig), the difference in i^**6**^A37 efficiency might reflect differential modification at another site(s) or some other feature(s). Related to this we note that all eukaryotic cy-tRNAs^**Trp**^CCA in the database representing mammals, chicken, plants and *S*. *cerevisiae*, contain 2'-*O*-methyl-cytidine (Cm34) at position 34 [83]. An encoded C34 in mt-tRNAs^**Trp**^ is found only in plants [83] and *Schizosaccharomyces* species [84]. The U34 of mt-tRNA^**Trp**^UCA in metazoan and many yeasts is hypermodified to decode numerous UGA (Trp) codons in their mt-mRNAs [83, 85, 86]. It was suggested that the wobble C34 of *S*. *pombe* mt-tRNA^**Trp**^CCA is modified to facilitate recognition of the UGA (Trp) codon found in mt *rps3* [84]. However, the only known modified C that can decode A is lysidine, which has so far been found in tRNAs^**Ile**^CUA [87]. We did not detect a difference at C34 of *S*. *pombe* cy- and mt- tRNAs^**Trp**^CCA by primer extension using low (0.01 mM) dNTPs [88].

### A Mod5-type restriction against CCA anticodon recognition appears inherent to TRIT1

Biological relevance of the exceptional absence of i^**6**^A37 modification of *S*. *cerevisiae* tRNA^**Trp**^CCA-A37-A38 had been enigmatic. Because human mt-tRNA^**Trp**^UCA is one of several mt-tRNAs with i^**6**^A37, it might have been unexpected that TRIT1 would exhibit strong substrate-specific restriction against *S*. *pombe* tRNA^**Trp**^CCA-A37-A38 (Fig 6) [43]. This is important because it is evidence that a restrictive type IPTase is not a peculiarity of Mod5. Further, it indicates that restriction toward a tRNA^**Trp**^CCA ACL is an IPTase characteristic in extant higher eukaryotes even though their cy-tRNAs^**Trp**^CCA contain G37. Yet, additional findings suggest that TRIT1 exhibits a restrictive type recognition that is conditional on features of the YYA ASL that is distinct from Mod5 type recognition. This raises possibilities for future studies of potentially new modes of ASL recognition.

### Insight into tRNA-i^6^A37 identity profiles of eukaryotes

Phylogenetic analysis of gene sequences encoding cy-tRNAs for UNN codons other than Ser in representative advanced animal species shows that G is uniformly present at position 37 as noted for Table 1. By contrast, primitive animals, *Dictyostelium discoidium* and *Strongylocentrotus purpuratus*, as well as yeasts *S*. *pombe*, *S*. *cerevisiae*, *Aspergillus* and *Candida* subspecies, have G or A at positions 37 of tRNAs^**Trp**^ and tRNAs^**Cys**^ [71, 89], also noted for Table 1, Fig 7 and S3 Fig. These data suggest that tRNAs-i^**6**^A37 identity was relatively variable in yeasts and primitive animal cells but became progressively less variable and fixed in higher eukaryotes, by patterns predominantly set by base identity of A or G at position 37.

We set an experiment to compare the IPTases from three divergent species, on *S*. *pombe* cy-tRNA^**Trp**^CCA-A37-A38 substrates that differ at one encoded nucleotide, C32 vs. U32, one of the differences between the *S*. *cerevisiae* and *S*. *pombe* cy-tRNAs^**Trp**^CCA-A37-A38. When the *S*. *pombe* cy-tRNA^**Trp**^CCA with either C32 or U32 was expressed in *S*. *cerevisiae* the results were consistent with our prior experiments in which the IPTases were compared in each of the two yeasts. The substrate-specific restriction observed for TRIT1 against *S*. *pombe* cy-tRNA^**Trp**^CCA -U32, was demonstrated in *S*. *cerevisiae* while this IPTase maintained efficient modification of the endogenous *S*. *cerevisiae* cy-tRNA^**Trp**^CCA. Moreover, the point mutation, U32C, converted the *S*. *pombe* cy-tRNA^**Trp**^CCA from a poor to a better substrate for TRIT1, while Tit1 modified both substrates to comparable efficiencies. The results also raised a new hypothesis, that another difference in the cy-tRNAs^**Trp**^CCA of the two yeasts, G26, may be responsible for the higher activity of Mod5 for the *S*. *pombe* cy-tRNA^**Trp**^CCA -C32, while remaining relatively inactive for the endogenous cy-tRNA^**Trp**^CCA which encodes U26 (S2 Fig, Fig 7B–7D). The hypothesis that modification of G26 to m^**2**^_**2**_G26 may promote i^6^A37 modification of certain tRNAs, including *S*. *pombe* cy-tRNA^**Trp**^CCA which carries both modifications [90] is plausible because m^**2**^_**2**_G26 can influence ASL structure [91]. This is a testable hypothesis fit for future study.

We note that our findings also have additional implications. Human mt-tRNA^**Trp**^UCA-A37-A38 carries U32 and U34. This and other observations suggest the possibility that the cmnm^**5**^U34 and tm^**5**^U34 modifications on *S*. *cerevisiae* and human mt-tRNAs^**Trp**^UCA [4, 83, 92] enhance activities of their substrate tRNAs for the respective IPTases. It is tempting to speculate that bulky modified uridines would also enhance other YYA AC IPTase substrates, the suppressors tRNA^**Tyr**^UUA, tRNA^**Ser**^UCA, and the tRNA^**Ser[Sec]**^UCA, which contain mcm^**5**^U (or hypermodified versions) [93–96].

## Materials and methods

***S*. *pombe* and *S*. *cerevisiae* yeast transformations** were by standard methods. All strains were published previously as noted. MT-8 (*mod5Δ*) lacks codons 1–250 of Mod5 and 35 bp upstream [34].

### DNA constructs

The different TRIT1 mutants and wild-type for mammalian transfection were cloned in the HindIII and AgeI sites of the pEGFP-N1 vector. A linker of 5 glycine residues was introduced between the TRIT1 sequences and GFP.

All primers and oligos were obtained from Eurofins Genomics (Tables 2 and 3). For *S*. *pombe*, Tit1 and Tit1-T12A, both with a C-terminal HA tag were expressed from the *tit1*^***+***^ promoter in a pRep vector in which the *nmt1*^***+***^ promoter was excised [43]. TRIT1 and Mod5 were expressed from the *nmt1*^***+***^ promoters in pRep82X or 4X, cloned such that their ATG immediately followed the Xho I site. Mutants were made using site-directed mutagenesis or PCR. The Mod5MTS-M12A-TRIT1 construct was generated by site directed mutagenesis using the Q5 *Site*-*Directed Mutagenesis* kit (New England Biolabs). All constructs were sequence verified.

**Table 2:** Oligo-DNAs used for PHA6 assay; ACL (anticodon loop), BP (body probe).

cy-tRNA-Tyr-GUA-BP (HS)	CGAGCCGGAATCGAACCAGCG
cy-tRNA-TrpCCA-A37-ACL (HS)	CCTAAGGATTTACAGTCCTCCGC
cy-tRNA-TrpCCA-G37-ACL (HS)	CCTAAGGATCTACAGTCCTCCGC
mt-TrpCCA-ACL (SC)	CCTAATGATTGATTTGAAATCAACTG
mt-TyrGUA-ACL (SC)	AAGTCATTGAGTTTACAGCTCAACT
cy-SerAGA-ACL (SC)	GCCCAAAAGATTTCTAATCTTTCG
cy-tRNA-Trp-T32 ACL (SP)	CTTTGAGATTTGGA**A**TCTCACAC
cy-tRNA-Trp-C32 ACL (SP)	CTTTGAGATTTGGA**G**TCTCACAC
mt-tRNATrp-UCA-ACL (SC)	CCTAATGATTGATTTGAAATCAACTG
mt-tRNATyr-GUA-ACL (SC)	AAGTCATTGAGTTTACAGCTCAACT
cy-tRNATrp-CCA-ACL (SC)	CAACCCTTCGATTTGGAGTCGAA
cy-tRNASer-AGA-ACL (SC)	GCCCAAAAGATTTCTAATCTTTCG
cy-tRNATyr-GUA-ACL (SC)	GATCTCAAGATTTACAGTCTTGC
mt-tRNATrp-UCA-BP (SC)	TAAAGAGATTCGAACTCCTAATG
mt-tRNATyr-GUA-BP (SC)	GGAATAGGAATTGAACCTATGAAG
cy-tRNATrp-CCA-BP (SC)	GACAGGAATTGAACCTGCAAC
cy-tRNASer-AGA-BP (SC)	CTGCAGGACTCGAACCTGC
cy-tRNATyr-GUA-BP (SC)	GGCGAGTCGAACGCCCGAT

**Table 3:** PCR Primers used in this study.

TRIT1F202L_Fwd	TCATAGTGAAcTTCTCCATCG
TRIT1F202L_Rev	GAGATTCCTGTTTCTTCAAAAAC
Mod5MTS-TRIT1_M12A_F	TTGCTTAAATgctTCTAAAAAAGTTATAGTGATTCTCGGG
Mod5MTS-TRIT1_M12A_R	CCTTTAAGCGGTCCCTTTAG
TRIT1_29-467F	CTCGGGGCCACGGGCAC
TRIT1_Xma1R	ATTACCCGGGTTAAACGCTGCATTTCAGCTC
TRIT1_Rev	ATATGGATCCTTAAACGCTGCATTTCAGCTCTTG
TRIT1_Fwd	ATTACTCGAGATGGCGTCCGTGGCGGCT
Xma1_Tit1_R	ATTACCCGGGTCATTTTAAAATTCCAATGTTTTTTAAACG
Xho1-TRIT1_R	ATATCTCGAGTTAAACGCTGCATTTCAGCTCTTG
Xho1-Tit11_R	ATATCTCGAGTCATTTTAAAATTCCAATGT
Xho1-Mod5_R	ATATCTCGAGTCATTCCACAGTCTCCTTCTTGTT
Nco1-TRIT1_F	ATTACCATGGTGGCGTCCGTGGCGGCT
Nco1_57-467TRIT1_F	ATATCCATGGTGCAGGTCTATGAAGGCCTAG
Xho1_57-467TRIT1_R	ATTACTCGAGTTAAACGCTGCATTTCAGCTCT
Nco1_TRIT1_Fwd	ATTACCATGGTGGCGTCCGTGGCGGCT
Xho1_TRIT1_Rev	ATATCTCGAGTTAAACGCTGCATTTCAGCTCTTG
Nco1_TRIT1 R17E/R21E_F	ATTACCATGGTGGCGTCCGTGGCGGCT
Xho1_TRIT1 R17E/R21E_R	ATATCTCGAGTTAAACGCTGCATTTCAGCTCTTG
HindIII_Tit1_F	GCAATCAAGCTTatcttataaaatgttaaagcccctttgcgttgta
XhoI_Tit1_R	AATTCCCTCGAGTCATTTTAAAATTCCAATGTTTTTTAAACGAATTTG
HindIII_Mod5_F	AGGATCAAGCTTAAAATCTTCATGCTAAAGGGACCGCTTAAAGG
Xho1_Mod5_R	AATTCCCTCGAGTCATTCCACAGTCTCCTTCTTGTTTAT
Nco1_TRIT1 T32A_F	ATTACCATGGTGGCGTCCGTGGCGGCT
Xho1_TRIT1 T32A_R	ATATCTCGAGTTAAACGCTGCATTTCAGCTCTTG
Nco1_TRIT1 57–467_F	ATATCCATGGTGCAGGTCTATGAAGGCCTAG
Xho1_TRIT1 57–467_R	ATTACTCGAGTTAAACGCTGCATTTCAGCTCT

*Cloning of IPTases in S*. *cerevisiae expression plasmids*. TRIT1, Tit1 and Mod5 ORF nucleotide sequences were PCR amplified using gene specific forward and reverse primers containing HindIII/Xho1 restriction sites. For Tit1 and Mod5, an existing ATG in pYX141 was disrupted prior to insertion of the ORF; pYX141 *(low copy*, *786 promoter)* was digested with EcoRI and BamHI, followed by treatment with Klenow fragment and ligation. Sequencing confirmed disruption of the ATG. The HindIII/Xho1 restriction sites were then used for ORF insertion. For TRIT1, TRIT1-R17E/R21E, TRIT1-T32A, and TRIT1(57–467), the sequences were PCR amplified from the corresponding pREPx plasmid constructs using primers that contained Nco1/Xho1 sites, and these were cloned into the corresponding sites of pYX141 or pYX242 (high copy, TP1 promoter).

*tRNA gene cloning*. HindIII fragments containing the human nuclear-encoded tRNA^**Tyr**^GUA gene 2–1 (TRA-GUA2-1) [71] with G37 or A37 and including 150 bp upstream and 90 bp downstream of the mature tRNA sequence [97] were synthesized by Genewiz (https://www.genewiz.com/), then cloned into the HindIII site of YCplac33 (CEN4). All DNA clones were confirmed by sequencing.

### Cell culture and transfection

HEK293 cells were maintained in DMEM plus Glutamax (Gibco) supplemented with 10% heat-inactivated FBS in humidified 37°C, 5% CO_2_ incubator. The cells were transfected using Lipofectamine 2000 according to manufacturer’s instructions using 2.5 ug plasmid DNA and 6.25 ul lipofectamine on cells that were 80% confluent in 6-well plates. The next day, cells were seeded onto coverslips for confocal microscopy (see below), or divided over multiple 10 cm plates for mitochondrial preparation (see below) and western blot analysis.

### Mitochondrial preparation and cell subfractionation

Subcellular fractions were isolated as described previously [98], with a few modifications. Forty-eight hours post transfection, HEK293 cells (8 x 10 cm plates at 80% confluency per condition) were washed with 10 ml warm PBS per plate and scraped in 5 ml ice-cold PBS per plate and combined in a 50 ml Falcon tube. Cells were spun down for 3 mins at 1200 rpm and the pellets were washed twice in 10 ml ice-cold PBS. An aliquot of cell pellet was kept for isolation of total protein in RIPA buffer (Pierce) containing protease inhibitors (Roche). Pellets were suspended in 2 ml of homogenization buffer (HB, 0.6 M Mannitol, 1 mM EGTA, 10 mM Tris-HCl pH 7.4) +0.1% BSA) and transferred to a hand-held 10 ml glass-Teflon homogenizer. After 25 strokes, the suspension was transferred to a 2 ml Eppendorf tube and centrifuged at 400g for 10 min at 4C. The supernatant, containing crude mitochondria was kept. The pellet was resuspended in 2 ml HB and homogenization was repeated using 15 strokes. Suspension was transferred to a 2 ml Eppendorf tube and centrifuged at 400g for 10 min at 4C. Again, the supernatant, containing crude mitochondria was kept. This process was repeated one more time with 15 strokes. All three supernatants were centrifuged again at 1000g for 10 mins at 4C. Supernatants were transferred into fresh Eppendorf tubes without disturbing the pellets and spun at 11,000g for 10 mins at 4C to pellet the mitochondria. The supernatant of the first homogenization was kept as the soluble cytoplasmic fraction. Mitochondria were washed twice with 1ml HB without BSA and PMSF and combined into one tube and centrifuged again at 11,000g for 5 mins at 4C to generate a single pellet. This was taken up in 500 ul of HB without BSA and PMSF and split into 2 tubes (200 ul/tube). One tube was put on ice (ProtK fraction) and the other one (mitochondrial fraction) was spun down and the pellet taken up in 40 ul RIPA buffer (Pierce) containing protease inhibitors (Roche). Protein concentrations of the total protein- and mitochondrial fractions were determined using BCA (Pierce). 40 ug of the protK fraction in a total of 190 ul HB without BSA and PMSF was treated with 1.6 ug proteinase K (Viagen) by incubating on ice for 15 mins and subsequently at room temperature for 15 mins. 5 mM PMSF was added to inactivate the Proteinase K and the mitochondria were spun down at 11,000g for 5 mins at 4C. Mitochondria were washed with 1 ml HB including PMSF, but without BSA and taken up in 40 ul RIPA buffer (Pierce) containing protease inhibitors (Roche). For the cytosolic fraction, equal protein concentration between wild-type and mutant was determined using nanodrop using HB as blank.17.5 ug of each fraction was separated on SDS-PAGE and analyzed on western blot.

### Western blots

Total protein was extracted from transfected HEK293 cells lysed in RIPA buffer (Pierce) containing protease inhibitors (Roche) 48 hours post transfection and size separated using SDS-PAGE. Proteins were then transferred to a nitrocellulose membrane. Antibodies used were anti-GFP (Santa Cruz, sc-8334), anti-Tom20 (Santa Cruz, sc-11415), anti-NDUFA9 (Abcam, ab14713) and polyclonal human anti-La (Go) was a gift of J. D. Keene and D. J. Kenan (Duke Medical Center) [99]. Anti-TRIT1 Ab was generated in rabbits using the peptide KLHPHDKRKVARSLQVFEE as an antigen and was affinity purified using the same peptide (Pierce protein research). Primary antibodies were detected by secondary antibodies from LI-COR Biosciences, which were conjugated to either IRDye 800CW or 680RD and western blot were scanned and quantified using the Odyssey CLx imaging system (LI-COR Biosciences).

Western blots for *S*. *pombe* protein: For protein isolation from *S*. *pombe*, cells were transferred to a 1.5 mL microcentrifuge tube, an equal volume of 0.7 N NaOH solution was added, mixed, then incubated at room temperature for 3 minutes. The tube was centrifuged at 5,000 rpm for one min and the supernatant discarded. SDS-PAGE sample buffer was added, mixed well and the sample was heated at 95°C for 5 min centrifuged again and the resulting supernatant recovered for analysis. Proteins were then transferred to a nitrocellulose membrane and subsequently analyzed. Anti-β-actin was from Abcam (mAbcam 8224) and used at a 1:5000 dilution. Blots were scanned as described above using the Odyssey CLx imaging system (LI-COR Biosciences).

### Confocal microscopy

24 hrs. after transfection cells were seeded onto coverslips. The next day the live cells were treated with MitoTracker (ThermoFisher) at 200 nM in PBS for 20 min. at 37°C. Cells were then fixed with 4% paraformaldehyde for 15 min. at room temperature and washed with PBS. PBS containing 1 ug/ml Hoechst 33342 (Sigma) was added and allowed to sit for 10 min, followed by a final PBS wash. Imaging was with a Leica DM IRE2 confocal microscope using a HCX PL APO CS 63.0x1.32 oil UV objective. Images were scanned in sequential mode in Hoechst, GFP and MitoTracker channels.

### PHA6 northern blotting

We recently found that increasing the post-hybridization wash temperature, to as high as 15°C above the Ti (hybridization incubation temperature, below) increased the difference in signal between IPTase-positive and IPTase-negative sample tRNAs, especially, mt-tRNAs. We note that the optimal wash temperature for ACL probes varies among individual tRNAs possibly due to other ACL modifications and other characteristics. We found that subjecting each tRNA-ACL probing to sequential washes at progressively higher temperatures yielded more reliable and higher apparent % modification efficiencies than subjecting all tRNA-ACL probe combinations to a final wash at the same temperature. After the maximum difference in IPTase-positive and -negative sample tRNAs is recorded, the next high temperature signal from all samples may substantially or drastically decrease. The results reported and quantified in this paper are from blot washings that reveal the greatest difference in ACL probe signal between IPTase positive and IPTase negative sample tRNAs that were obtained using this approach. After the maximum difference in IPTase-positive and -negative sample tRNAs occurs, the next high temperature wash may drastically decrease signal from all samples and may not be useful for quantification.

Novex 10% TBE-urea polyacrylamide gels and transferred to GeneScreen plus nylon membranes using Invitrogen iBlot transfer apparatus. Blots are UV crosslinked then baked in a vacuum oven at 80°C for 1–2 hrs. The blot is incubated in 10–15 ml of PHA6 hybridization buffer contains 2× SSC (1× SSC is 0.15 M NaCl plus 0.015 M sodium citrate) plus 0.2% SDS, 1× Denhardt's solution [Life Technologies], 100 μg/ml sheared salmon sperm DNA (supplier) at the oligonucleotide DNA probe hybridization temperature for 1 to 2 h. A ^**32**^P-end-labeled antisense DNA oligonucleotide at >10^8^ cpm/μg DNA was added to the hybridization buffer (final concentration, 10^5^ cpm/ml) and left overnight for hybridization at the Ti. The Ti is calculated as follows: Ti = *T*_m_—15°C where *T*_m_ is equal to 16.6 log[M] + 0.41[*P*_gc_] + 81.5 − *P*_*m*_ − (*B/L*) − 0.65, where M is the molar salt concentration, *P*_gc_ is the percent G+C content in the oligonucleotide DNA probe, *P*_*m*_ is the percentage of mismatched bases, if any, *B* is 675, and *L* is the oligonucleotide DNA probe length [100]. Blots were washed with 2× SSC, 0.2% SDS two times at room temperature (10 min each) followed by 30 min at Ti +5°C, +7.5°C, +10°C, +12°C or +15°C (determined empirically, described above). Optimal wash temperatures for different tRNAs has ranged from Ti to T1+15°C.

Before the next hybridization, blots were stripped with 0.1 × SSC, 0.1% SDS at 85–90°C, and the nearly complete removal (≥90%) of ^32^P was confirmed by Phosphor Imager analysis. In general, multiple ACL probings are done followed by the body probes. Table 2 provides sequences of the oligo-DNAs used as ACL and body probes.

The formula used to calculate % i^**6**^A37 modification is [1-(ACL*tit1-*Δ (transformed with IPTase constructs)/BP*tit1-*Δ (transformed IPTase constructs))/(ACL*tit1*Δ (empty vector)/BP*tit1*Δ (empty vector))] x 100, where % modification in yNB5 (*tit1-Δ*) = 0, “ACL” indicates the ACL probe and “BP” indicates the body probe. In cases where MT-8 cells were used rather than yNB5, *mod5*Δ would substitute for *tit1*Δ. As described, the formula is internally normalized by the *tit1*Δ or *mod5*Δ samples.

## Supporting information

S1 Fig Concentration-dependent i^6^A37 modification of cy-tRNA^Trp^CCA. **A)** Translation initiation contexts of ATG codons; the first two lines show consensuses derived for *S*. *cerevisiae* [highly expressed genes, refs 101, 102] and the last line shows a consensus derived for vertebrate [103]. Lines 3 and 4 are for the alternative ATGs that encode MOD5 M1 and M12 in their native context; lines 5 and 6 are for the MOD5 M1 and TRIT1 M1 after cloning into the *Xho1* site of *S*. *pombe* expression vectors pRep4X or pRep82X (*pRepXho1*). Line 7 shows the native TRIT1 M1 ATG context. **B)** Nucleotide sequence of MOD5 including its M1 and M12 ATG sites and the M12A substitution mutation in the Mod5MTS-M12A-TRIT1 construct. **C)** Western blot of TRIT1 protein developed using anti-TRIT1 Ab, from cells with constructs containing the strong (pRep4X) or weak (pRep82X) *nmt1*^***+***^ promoter (see text), indicated above the lanes as 4X or 82X. Lanes are numbered below. MW markers are indicated in kDa. The shared band below 25 kDa is an internal control. Lower panel shows β-actin as loading control used for quantitative normalization. **D)** Determination of TRIT1 levels in C by quantitative Odyssey CLx imaging (Methods); numbers above bars indicate TRIT1/β-actin levels in each sample; The pRep vectors used are indicated as 4X and 82X are indicated along the X-axis. **E)** Northern blot of 2 cy- and 2 mt- tRNAs by TRIT1 and Mod5MTS-TRIT1 each from the strong promoter, pRep4X and weak promoter pRep82X, as indicated above the lanes, as 4X and 82X. The top four panels show the ACL probings as indicated to the left, and the bottom four panels show the corresponding body probings. **F)** Quantitation of % i^**6**^A37 modification of the mt-tRNAs and the cy-tRNAs; the pRep vectors used are indicated as 4X and 82X along the X-axis. **G)** Clover leaf representations of *S*. *pombe* cy-tRNA^**Trp**^CCA and mt-tRNA^**Trp**^CCA as encoded by the nuclear and mitochondrial DNA and folded by tRNAscan-SE [112] (Table 1, Discussion).
(TIF)

S2 Fig Clover leaf structures predicted by tRNAscan-SE for the cy-tRNAs^Trp^CCA of *S*. *pombe* and *S*. *cerevisiae* [ref 112]. (TIF)

S3 Fig Sequence alignments of the ACLs of cy-tRNA^**Trp**^CCA (A), cy-tRNA^**Cys**^GCA (B), and cy-tRNA^**Tyr**^GUA (C), in the eukaryotes indicated; the 32 and 37 positions are numbered and the horizontal bar indicates the AC. The empty boxes reflect that no genes for this tRNA were indicated for this species [ref 112].
(TIF)
