# Supplementary figures and images for: Targeting mitochondrial and cytosolic substrates of TRIT1 isopentenyltransferase: Specificity determinants and tRNA-i6A37 profiles
Source: PLoS Genet. 2020 Apr 23;16(4):e1008330. doi: 10.1371/journal.pgen.1008330 (PMC7200024; doi:10.1371/journal.pgen.1008330)

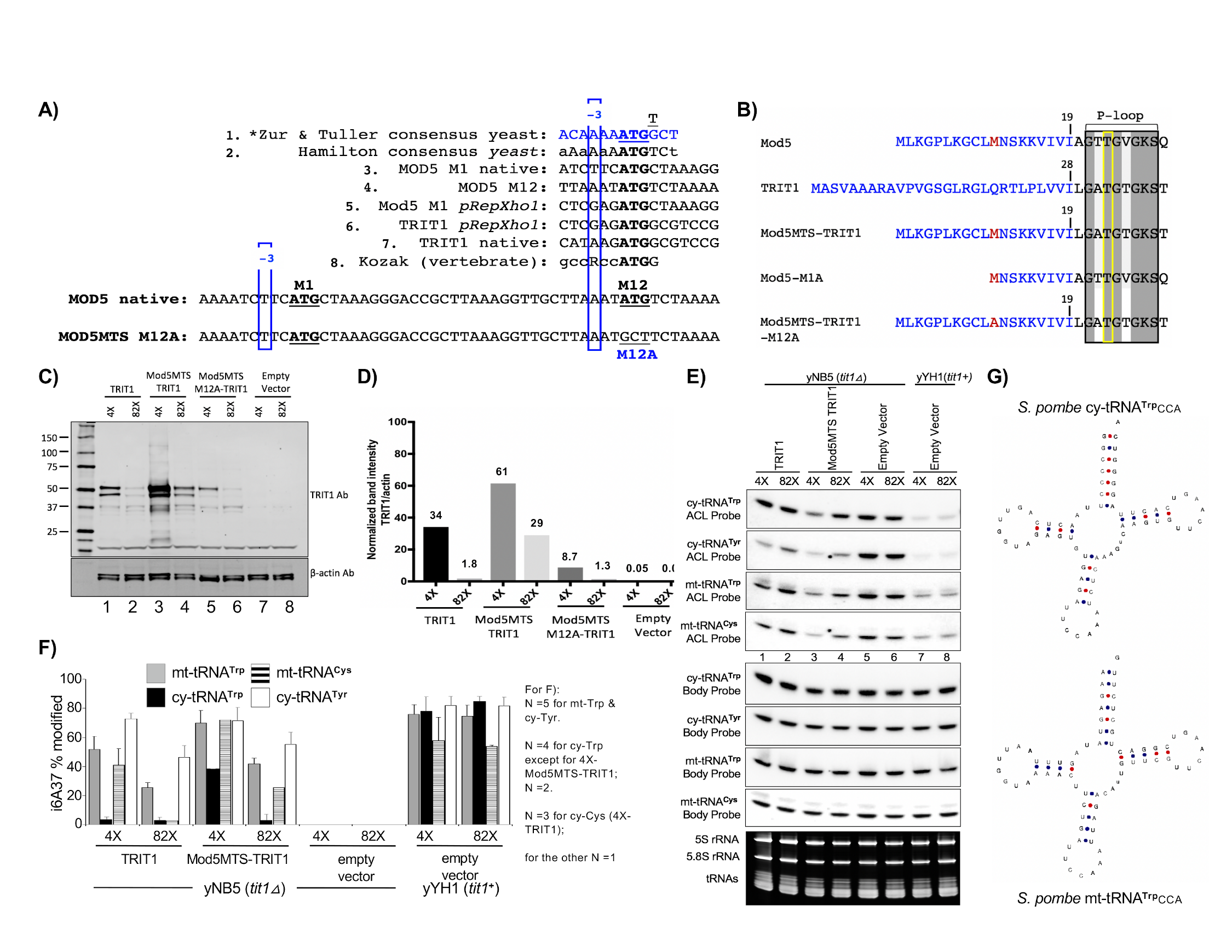

Supplement: S1 Fig — A) Translation initiation contexts of ATG codons; the first two lines show consensuses derived for S. cerevisiae [highly expressed genes, refs 101, 102] and the last line shows a consensus derived for vertebrate [103]. Lines 3 and 4 are for the alternative ATGs that encode MOD5 M1 and M12 in their native context; lines 5 and 6 are for the MOD5 M1 and TRIT1 M1 after cloning into the Xho1 site of S. pombe expression vectors pRep4X or pRep82X (pRepXho1). Line 7 shows the native TRIT1 M1 ATG context. B) Nucleotide sequence of MOD5 including its M1 and M12 ATG sites and the M12A substitution mutation in the Mod5MTS-M12A-TRIT1 construct. C) Western blot of TRIT1 protein developed using anti-TRIT1 Ab, from cells with constructs containing the strong (pRep4X) or weak (pRep82X) nmt1+ promoter (see text), indicated above the lanes as 4X or 82X. Lanes are numbered below. MW markers are indicated in kDa. The shared band below 25 kDa is an internal control. Lower panel shows β-actin as loading control used for quantitative normalization. D) Determination of TRIT1 levels in C by quantitative Odyssey CLx imaging (Methods); numbers above bars indicate TRIT1/β-actin levels in each sample; The pRep vectors used are indicated as 4X and 82X are indicated along the X-axis. E) Northern blot of 2 cy- and 2 mt- tRNAs by TRIT1 and Mod5MTS-TRIT1 each from the strong promoter, pRep4X and weak promoter pRep82X, as indicated above the lanes, as 4X and 82X. The top four panels show the ACL probings as indicated to the left, and the bottom four panels show the corresponding body probings. F) Quantitation of % i6A37 modification of the mt-tRNAs and the cy-tRNAs; the pRep vectors used are indicated as 4X and 82X along the X-axis. G) Clover leaf representations of S. pombe cy-tRNATrpCCA and mt-tRNATrpCCA as encoded by the nuclear and mitochondrial DNA and folded by tRNAscan-SE [112] (Table 1, Discussion). (TIF) [file pgen.1008330.s001.tif]

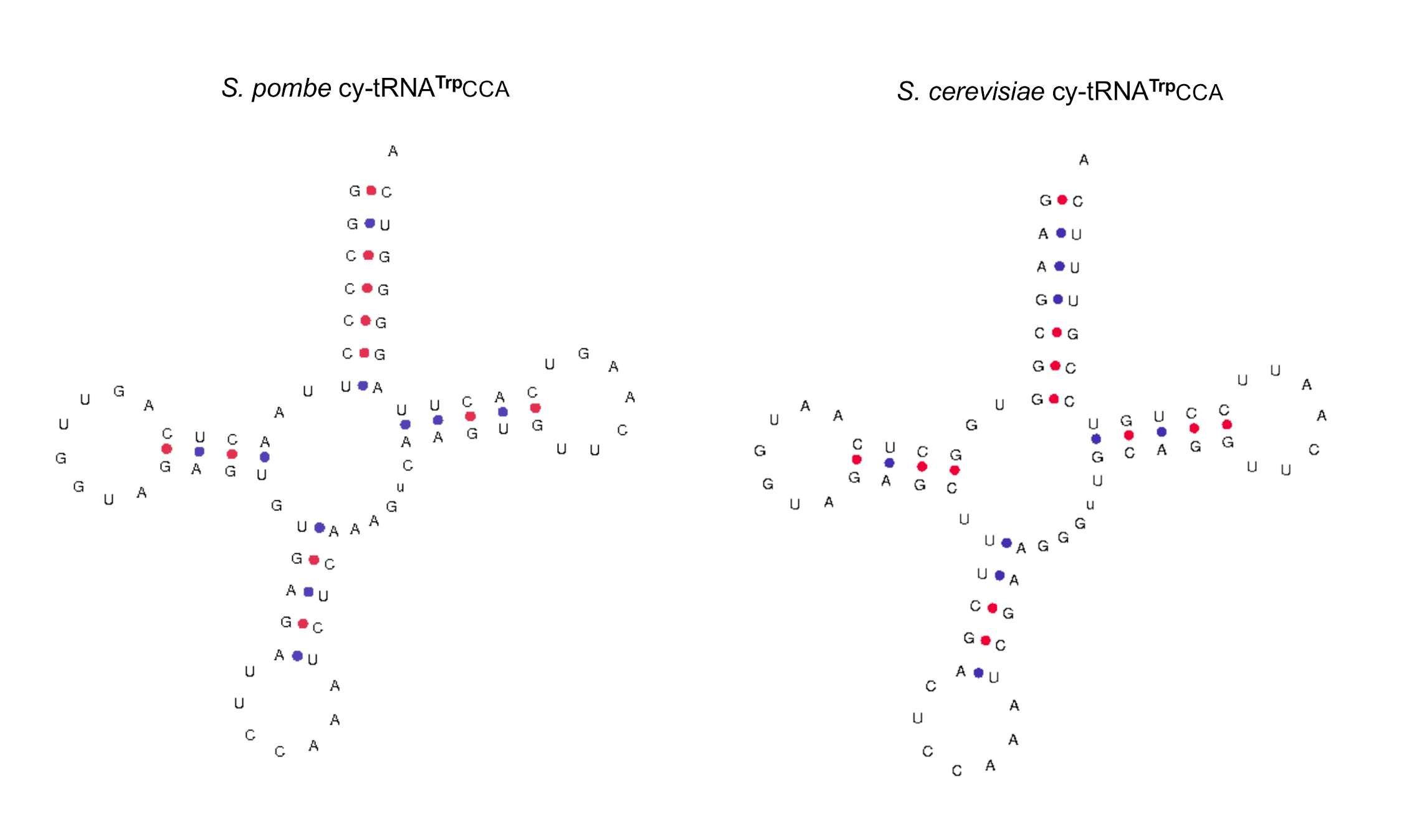

Supplement: S2 Fig — (TIF) [file pgen.1008330.s002.tif]

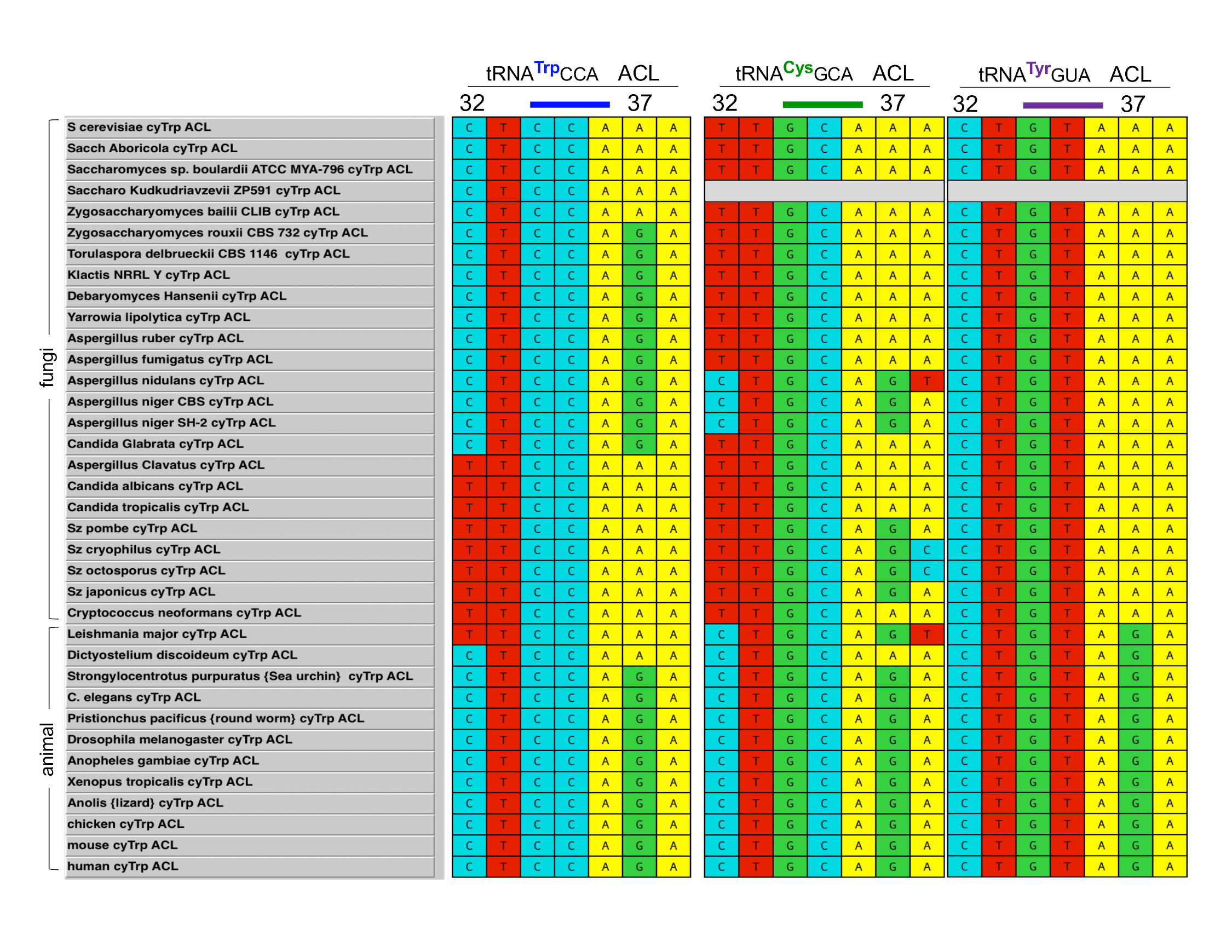

Supplement: S3 Fig — Sequence alignments of the ACLs of cy-tRNATrpCCA (A), cy-tRNACysGCA (B), and cy-tRNATyrGUA (C), in the eukaryotes indicated; the 32 and 37 positions are numbered and the horizontal bar indicates the AC. The empty boxes reflect that no genes for this tRNA were indicated for this species [ref 112]. (TIF) [file pgen.1008330.s003.tif]
